# ResViT-GANNet: a deep learning framework for classifying breast cancer histopathology images using multimodal attention and GAN-based augmentation

**DOI:** 10.1186/s12880-025-01940-6

**Published:** 2025-09-29

**Authors:** Yuee Zhou, Fengqing Jin, Guodong Suo, Jianlan Yang

**Affiliations:** 1 School of Medical Information Engineering, Gansu University of Chinese Medicine, Lanzhou, Gansu 730000 China; 2 Quanzhou Orthopedic Traumatological Hospital, Quanzhou, Fujian 362000 China

**Keywords:** Breast cancer classification, Histopathological image, ResNet, Vision transformer, Generative adversarial network, Multimodal attention mechanism

## Abstract

**Background:**

Breast cancer remains the most commonly diagnosed malignancy among women worldwide. Histopathological image analysis is the clinical gold standard for diagnosis; however, the high resolution and complexity of these images, together with limited annotated data, pose significant challenges for traditional deep learning methods. This study aims to develop a robust classification framework capable of effectively analyzing high-resolution histopathological images.

**Methods:**

We propose ResViT-GANNet, a novel dual-branch deep learning architecture that integrates a residual convolutional network with channel attention and a vision transformer with multi-layer token fusion. This design is specifically intended to capture both fine-grained local pathological features and long-range global semantic representations. A key novelty of our framework is the Token-Aligned Multimodal Attention (TAMA) module, which combines heterogeneous features from both branches through multi-head attention and token-wise alignment. To address limited and imbalanced data, we incorporated synthetic histopathology images generated with StyleGAN2-ADA into the training set. Extensive experiments on the BACH and BreakHis datasets demonstrate superior performance, with statistical significance confirmed through rigorous evaluation.

**Results:**

On the BACH dataset (4-class classification), ResViT-GANNet achieved an accuracy of 96.40%, precision of 96.34%, recall of 96.36%, and an F1-score of 96.35%. These results significantly outperformed baseline methods including TransMIL (85.83%), CTransPath (88.75%), and SwinCNN (92.89%), with p-values < 0.01 and large effect sizes (Cohen’s d > 1.0). Incorporating synthetic data yielded an average accuracy improvement of 3.3%. On the BreakHis dataset (8-class classification across four magnification levels), the model attained an average accuracy of 98.22%, with per-class accuracies ranging from 97.25% to 99.50%. Grad-CAM visualizations further confirmed enhanced interpretability and highlighted critical histological features relevant for classification.

**Conclusions:**

ResViT-GANNet substantially improves classification performance on complex, high-resolution histopathology images. The major contributions of this work include a parallel dual-branch architecture enabling synergistic local–global feature learning, a token-aligned multimodal fusion mechanism, and the integration of generative augmentation with explainable AI. Together, these innovations enhance model generalization and robustness, underscoring the potential of ResViT-GANNet as a clinically useful decision-support system for breast cancer diagnosis.

**Trial registration:**

Not applicable.

## Introduction

Breast cancer remains the most common malignancy worldwide, with 2.296 million new cases and 666,000 deaths reported in 2024 [1]. While early-stage cases are curable, survival rates decline dramatically in advanced stages, highlighting the need for early and accurate diagnosis. Histopathology is the clinical gold standard, where pathologists meticulously analyze high-resolution whole slide images (WSIs) to identify malignant tissue based on complex cellular and architectural patterns. However, this process is time-consuming, labor-intensive, subjective, and susceptible to interobserver variability. These limitations highlight the urgent need for advanced computational tools that can enhance pathological diagnosis, improve accuracy and efficiency, and reduce the growing burden on healthcare systems.

Deep learning methodologies have emerged as transformative tools in medical image analysis. Convolutional Neural Networks (CNNs), particularly architectures such as ResNet [2] and DenseNet [3], have become the cornerstone for breast cancer histopathology classification by effectively capturing localized cellular features (e.g., nuclear morphology, chromatin patterns) without relying on hand-crafted features. Significant efforts have been made to enhance CNN performance, including the integration of attention mechanisms to focus on diagnostically relevant regions [4], the use of multi-scale feature extraction to capture heterogeneous structures [5], and the incorporation of squeeze-and-excitation (SE) blocks for channel-wise feature recalibration [6]. The continuous pursuit of improved performance has led to the development of sophisticated ensemble and optimization strategies, including multi-CNN frameworks with grid-search optimization [7] and advanced meta-heuristic algorithms such as Differential Evolution and Salp Swarm Algorithm for weight tuning [8, 9]. Notably, frameworks like MSMV-PFENet [10] demonstrate the potential of parallel multi-stream CNNs. However, despite these advancements, a fundamental limitation persists: CNNs’ local receptive fields inherently constrain their ability to model long-range dependencies and global tissue-level contexts, which are crucial for comprehensive diagnosis.

The emergence of Transformer-based models has introduced a paradigm shift by leveraging self-attention mechanisms to capture global contextual information [11, 12]. Models such as Vision Transformer (ViT) and Swin Transformer have shown considerable success in various medical imaging tasks [13]. Recent research has further adapted them for histopathology-for example, by integrating them with deconvolutional networks for enhanced localization [14], employing multi-view encoders for richer context, or utilizing sliding-window mechanisms to better handle large images [15]. Furthermore, lightweight CNN-Transformer hybrids [16] and federated learning frameworks [17] have been investigated. A dominant trend has been the development of serial hybrid architectures. However, these approaches often compel CNN-extracted features to undergo irreversible compression (e.g., through global average pooling) before being processed by the Transformer, resulting in the loss of fine-grained details crucial for discriminating subtle pathological subtypes [18, 19]. Additionally, Transformers may still overlook subtle lesion morphology, often require large datasets for effective generalization, and many methods prioritize performance metrics over pathology-grounded interpretability, which is essential for clinical trust and adoption [20].

The unique characteristics of breast cancer histopathology images—including extremely high resolution, the necessity for multi-scale analysis (from nuclear atypia to tissue architecture), and subtle morphological differences between subtypes—significantly amplify these limitations. This convergence of challenges creates a pronounced research gap, urgently requiring a tailored framework that can simultaneously (a) seamlessly integrate local cellular details with global contextual information without feature loss, (b) incorporate pathology-specific prior knowledge to guide feature learning and fusion, and (c) mitigate data scarcity while providing transparent, clinically verifiable explanations.

To address these challenges, we propose ResViT-GANNet, a novel CNN–Transformer hybrid specifically designed for breast cancer pathological image classification. Our main contributions are:

1. **Parallel dual-branch CNN–Transformer architecture with cross-modal feature fusion.** Unlike conventional serial CNN–Transformer models where feature loss is irreversible, our design processes the input through parallel CNN and ViT branches, capturing both cellular-level local textures and tissue-level global structures. Features from the two branches are explicitly integrated using the Token-Aligned Multimodal Attention (TAMA) module, which aligns feature spaces, suppresses redundancy, and enhances complementarity. This architecture improves robustness and discriminative capability across breast cancer subtypes.
2. **Pathology-specific feature optimization with multi-scale representation.** We systematically evaluate attention mechanisms and demonstrate that channel recalibration (SE) effectively emphasizes tumor-relevant features while suppressing irrelevant background, outperforming spatial or hybrid attention. Furthermore, the ViT branch aggregates multi-layer [CLS] tokens to capture hierarchical features at cellular, glandular, and tissue levels, improving recognition of challenging subtypes such as in situ carcinoma and enabling the model to better mirror pathologists’ multi-scale reasoning.
3. **Data augmentation and enhanced model interpretability.** To overcome limited dataset sizes, we generate high-resolution, class-controlled synthetic images using StyleGAN2-ADA, expanding training diversity and improving accuracy and F1-scores. In parallel, we enhance clinical trust through Grad-CAM visualization, confirming that the model focuses on pathologically relevant regions such as ductal disruption and nuclear density, thereby increasing transparency and confidence in decision-making.

Collectively, our work integrates pathology-aware feature modeling, multi-scale learning, data augmentation, and interpretability into a unified framework, advancing breast cancer histopathology image classification. Rigorous evaluation on the BACH and BreakHis benchmarks shows that our method achieves superior performance with clinically interpretable predictions. By combining robust augmentation and explainable AI within a dedicated architecture, this study offers a reliable and practical step toward AI-assisted clinical diagnosis, effectively bridging advanced computational power with clinical practice.

## Related work

The development of automated classification methods for breast cancer histopathology images has evolved through several distinct phases, primarily driven by CNN-based, Transformer-based, and hybrid approaches.

### CNN-based breast cancer classification methods

Convolutional neural networks (CNNs) effectively model local image structures through mechanisms such as receptive fields, parameter sharing, and downsampling. Discriminative features are automatically extracted via hierarchical convolution and pooling operations, eliminating the need for manually crafted prior knowledge. Classic CNN architectures such as ResNet, VGG, DenseNet, and Xception have been widely adopted for histopathological image analysis in breast cancer diagnosis.When combined with attention mechanisms and residual connections, these models demonstrate enhanced capacity to distinguish heterogeneous tissue structures, leading to notable improvements in classification performance. For example, Yang [4] et al. proposed a CNN integrated with a continuous attention mechanism that emphasizes diagnostic regions in pathology images through ROI supervision; however, the model exhibited limited generalization capability. Jiang [6] et al. combined residual modules with SE blocks, while Xie [5] et al. developed an automated multiscale end-to-end deep classification network—both of which leveraged multi-scale features to enhance classification accuracy. Notably, Liu [10] et al.‘s MSMV-PFENet achieved promising performance on the BACH dataset by employing three parallel CNNs for feature extraction. However, due to the limited number of annotated training samples, the model suffered from overfitting and demonstrated reduced robustness to outliers. Despite these advancements, CNN-based approaches inherently focus on local receptive fields, which restricts their ability to capture global contextual relationships within histopathological images. Given the spatial dependencies and morphological variability across different tissue regions, relying solely on local convolutions and pooling operations makes it difficult to model long-range interactions, thereby constraining overall discriminative power.

### Transformer-based breast cancer classification methods

Unlike convolutional neural networks (CNNs), Transformers are capable of capturing long-range dependencies between arbitrary positions in an image through the self-attention mechanism. This capability facilitates a more comprehensive understanding of global contextual relationships in histopathological images and enhances the model’s ability to perceive cross-regional tissue structures. In practical applications, a range of Transformer-based architectures has been proposed to address the inherent complexity of histopathological image analysis. For example, He [14] et al. combined DeConvNet with Transformer modules by leveraging self-attention to align hierarchical edge-detection (HED) channel information extracted via deconvolution. Tummala [13] et al. integrated multiple Swin Transformer variants, exploiting their complementary strengths to boost overall classification accuracy. Sreelekshmi [15] et al. utilized the Swin Transformer with a sliding-window mechanism to compute localized self-attention and aggregate cross-window dependencies, leading to improved global pattern recognition, particularly in large-scale images.Despite these advancements, Transformer-based models still face several limitations. Their computational complexity is significantly higher than that of CNNs, and they may struggle to accurately detect small lesions—an essential requirement in histopathological analysis. To mitigate these issues, recent studies have increasingly employed strategies such as transfer learning, self-supervised learning, and hybrid architectures that fuse Transformer modules with convolutional backbones. These approaches aim to improve convergence speed, computational efficiency, and robustness, particularly in data-constrained and high-resolution medical imaging scenarios.

### Hybrid-model-based breast cancer classification methods

Histopathological images are typically characterized by high resolution and complex structural patterns, making it challenging for a single model to comprehensively capture both deep semantic information and discriminative features. To enhance feature extraction and improve classification performance, researchers have increasingly explored model fusion strategies that integrate the strengths of multiple high-performing architectures. For example, transfer learning was employed to train six different CNNs, extract multi-layer features, and identify ResNet50 and Inception_v3 as the best-performing models based on validation loss. These two models were subsequently fused to boost overall classification accuracy. However, as fusion models often increase computational complexity and parameter overhead, some studies have focused on lightweight network design as an optimization goal. For instance, Yi [21] et al. proposed IDC-Net, a lightweight model that combines the representational strength of CNNs and Capsule Networks (CapsNet), significantly reducing parameter count and computational cost while maintaining high classification performance.In addition, given that CNNs excel at capturing local spatial features, whereas Transformers are more adept at modeling long-range dependencies, recent research has attempted to combine both paradigms to enable joint modeling of local and global information.

In summary, while existing methods have laid a solid foundation, the challenge of fully extracting and efficiently fusing multi-scale features without information loss remains central to histopathological image analysis. Most hybrid models adopt a serial pipeline that inevitably compresses fine-grained CNN features before global Transformer processing. Unlike these approaches, our proposed ResViT-GANNet introduces a parallel dual-branch architecture designed to circumvent this fundamental limitation. Furthermore, our novel Token-Aligned Multimodal Attention (TAMA) module explicitly addresses feature misalignment between branches, while our integrated use of generative augmentation tackles data scarcity—challenges not comprehensively addressed in prior works.

The Table 1 systematically compares the main methods, highlights their specific limitations (e.g., serial processing leading to feature loss, ignoring global context, not paying attention to pathology priors), and clearly illustrates how the innovations in ResViT-GANNet (e.g., parallel two-branch design, TAMA module, pathology-specific SE) overcome these challenges.

**Table 1:** Comparative analysis of recent studies and limitations addressed by ResViT-GANNet

Study	Key Methodology	Identified Limitations	How ResViT-GANNet Addresses This
TransMIL [27]	Transformer for Multiple Instance Learning on WSI bags.	Processes instance sequences, may lose fine-grained inter-patch relationships.	Parallel dual-branch design preserves full-resolution local features alongside global ViT features.
Swin Transformer	Hierarchical Transformer with shifted windows.	Can struggle with very fine-grained nuclear morphology without a strong CNN backbone.	Complemented by a dedicated ResNet branch specifically engineered to capture local cellular features.
SwinCNN [15]	Serial fusion of Swin Transformer and CNN features.	Serial architecture: CNN features are compressed before Transformer processing, losing details.	Parallel architecture avoids irreversible feature compression, allowing for lossless feature fusion.
CTransNet [28]	DenseNet backbone with weighted feature fusion strategy.	Fundamentally a CNN with feature optimization, lacks powerful global context modeling of Transformers.	Integrates a full Vision Transformer branch to capture hierarchical global semantics.
DWNAT-Net [30]	Combines Discrete Wavelet Transform (DWT) and Neighborhood Attention.	The handcrafted frequency decomposition (DWT) may not be optimal for all pathological features.	Learns features end-to-end from raw pixels, allowing the model to discover optimal representations.
FCCS-Net [31]	Fully convolutional network with channel and spatial attention.	As a fully convolutional network, it remains inherently local.	The ViT branch provides a global receptive field from the first layer, complementing local FCNN features.
MA-MIDN [32]	Multi-view attention for lesion detection and diagnosis.	Relies on multiple CNNs, computationally heavy yet still limited to local features.	A single, efficient parallel framework achieves both local and global feature learning.
MSMV-PFENet [22]	Parallel multi-stream CNNs with progressive feature coding.	Limited to CNN-level receptive fields, cannot model long-range tissue-level dependencies.	The ViT branch inherently captures long-range global contexts through self-attention.
ResViT-GANNet (Ours)	Parallel ResNet-50 & ViT with TAMA fusion and StyleGAN2-ADA.	N/A	Uniquely integrates a parallel dual-path design, pathology-optimized attention, hierarchical token fusion, and generative augmentation into a unified, end-to-end framework.

## Materials and methods

### Overall experimental workflow

The overall experimental workflow of this study was designed to ensure robustness and reproducibility, comprising four key stages:

1. **Data preparation and preprocessing** (Sect. “Materials and methods”), including normalization, augmentation, and rigorous partitioning of raw histopathology images into training, validation, and test sets;
2. **Model development and training** (Sect. “Research methodology”), involving forward propagation, loss computation, and backpropagation for parameter optimization within the proposed dual-branch ResViT-GANNet architecture;
3. **Model evaluation and experimental analysis** (Sect. “Results”), employing a comprehensive set of performance metrics and statistical tests to assess the trained model on a held-out test set; and.
4. **Qualitative analysis with explainable AI** (Sect. “Qualitative analysis with Grad-CAM visualizations”), using Grad-CAM visualizations to interpret model predictions and conduct error analysis.

This structured, end-to-end workflow provides a systematic and transparent pipeline from raw data input to final prediction and interpretation.

### Dataset

In this study, two publicly available breast histopathology image datasets, BACH [24] and BreakHis [25], were employed to evaluate the proposed model.

The **BACH dataset** comprises 400 H&E-stained images, evenly distributed across four subtypes: Normal (100), Benign (100), InSitu Carcinoma (100), and Invasive Carcinoma (100). All images are high-resolution RGB images with dimensions 2048 × 1536 pixels, acquired at a magnification of 200×, with a physical pixel size of 0.42 μm × 0.42 μm, and stored in uncompressed TIFF format. The dataset does not provide patient IDs or demographic information, which limits the possibility of patient-level splitting. Following common practice, data partitioning was performed at the image level.

The BreakHis dataset contains 7,909 images from 82 patients (24 benign, 58 malignant). It is divided into benign (2,368 images) and malignant (5,429 images) groups, with further subdivision into eight histological subtypes:

Benign: Adenosis (444), Fibroadenoma (1,014), Tubular Adenoma (453), Phyllodes Tumor (569).

Malignant: Ductal Carcinoma (3,451), Lobular Carcinoma (626), Mucinous Carcinoma (792), Papillary Carcinoma (560).

All images are RGB PNG format with a resolution of 700 × 460 pixels, captured at four magnification factors (40×, 100×, 200×, 400×). Unlike BACH, BreakHis provides patient IDs, enabling rigorous patient-level data splitting.

Table 2 presents the detailed distribution of the BreakHis dataset across different magnification factors (40×, 100×, 200×, and 400×) and histological subtypes. The dataset includes four benign tumor subtypes—Adenosis (A), Fibroadenoma (F), Tubular Adenoma (TA), and Phyllodes Tumor (PT)—and four malignant tumor subtypes—Ductal Carcinoma (DC), Lobular Carcinoma (LC), Mucinous Carcinoma (MC), and Papillary Carcinoma (PC). As shown in the table, the image counts vary substantially across magnifications and subtypes, leading to class imbalance and posing challenges for model training and evaluation.

**Table 2:** Distribution of BreakHis dataset by magnification and tumor subtype

Magnification	A	F	TA	PT	DC	LC	MC	PC	Total
40×	114	253	109	149	864	156	205	145	1995
100×	113	260	121	150	903	170	222	142	2081
200×	111	264	108	140	896	163	196	135	2013
400×	106	237	115	130	788	137	169	138	1820
Total	444	1014	453	569	3451	626	792	560	7909
Patients	4	10	3	7	38	5	9	6	82

### Dataset preprocessing

For the BACH dataset, all original TIFF images were first converted into 8-bit RGB format to standardize the data and ensure compatibility with the model input requirements. Each whole-slide image, with a native resolution of 2048 × 1536 pixels, was subsequently partitioned into 12 non-overlapping patches of 512 × 512 pixels using a fixed 4 × 3 grid strategy. This procedure ensured complete coverage of the tissue region while reducing the input size to a scale more suitable for deep neural networks, thereby enhancing computational efficiency. Nevertheless, the rigid grid-based partitioning may occasionally introduce redundancy or fail to capture diagnostically subtle regions compared to more adaptive patch-extraction approaches.

To further improve data diversity and model generalization, standard data augmentation techniques were applied during training, including random horizontal and vertical flips, random rotations, brightness and contrast adjustments, and Gaussian blurring. In addition, a generative adversarial network (StyleGAN2-ADA) was employed to synthesize 100 additional images for each of the four classes, thereby expanding the dataset to 800 images in total, with 200 samples per class.

Finally, the augmented dataset was randomly divided at the image level into training (70%), validation (10%), and test (20%) subsets. The detailed distribution is provided in Table 3. Although image-level splitting is a widely adopted practice for the BACH dataset due to the absence of patient identifiers, it is important to acknowledge that this approach cannot completely eliminate potential correlations between subsets. Such risks could be more effectively mitigated if patient-level metadata were available.

**Table 3:** Data split of the augmented BACH dataset

Class	Train (70%)	Validation (10%)	Test (20%)	Total
Normal	140	20	40	200
Benign	140	20	40	200
InSitu	140	20	40	200
Invasive	140	20	40	200
Total	560	80	160	800

To address the critical issue of class imbalance and rigorously prevent patient-level data leakage, we implemented a robust two-layer stratified five-fold cross-validation strategy for the BreakHis dataset, leveraging the available patient identifiers. The dataset exhibits substantial imbalance both at the macro level (Benign: 2,480 images; Malignant: 5,429 images) and across the eight fine-grained histological subtypes (e.g., Ductal Carcinoma: 3,451 images; Lobular Carcinoma: 626 images). A simple random split at the image level could yield subsets with unrepresentative class distributions, thereby biasing model training and compromising evaluation reliability.

To mitigate these issues, all images were first preprocessed by resizing to a consistent resolution of 700 × 460 pixels and converting to RGB format to standardize input dimensions and color encoding. To enrich local feature representations and enhance the model’s ability to capture discriminative morphological details, a sliding-window approach was employed to extract overlapping 224 × 224 patches with a stride of 112 pixels. This strategy substantially increased the number of training samples and facilitated multi-scale contextual learning. Although patch overlap introduces some redundancy, this is offset by improved modeling of local tissue heterogeneity, which is particularly valuable in histopathological image analysis.

During training, additional data augmentation was applied, including random horizontal and vertical flips and small-angle rotations (± 10°), to improve invariance to orientation and morphological variability.

For dataset partitioning, we adopted a two-stage stratified group-splitting strategy via the StratifiedGroupKFold method. First, the dataset was partitioned into five folds at the patient level, ensuring that all images from a given patient were assigned exclusively to one fold. Then, each training-validation fold was further subdivided into training and validation subsets, again preserving patient boundaries. This approach provided two key advantages: (1) stratification maintained class distributions across all subsets, mitigating class imbalance, and (2) group-wise partitioning eliminated patient-level data leakage, ensuring that evaluation reflected model generalization to entirely unseen patients.

The five-fold cross-validation framework further improved the reliability of performance estimates by aggregating results across multiple folds, thereby reducing variability due to partitioning randomness. All extracted patches were stored along with their corresponding metadata—including patient ID, magnification factor, and class label—resulting in a structured and reusable dataset for model training. The detailed distribution is provided in Table 4.

**Table 4:** Detailed distribution of BreakHis dataset across splits (One Fold)

Subtype	Total	Training Set	Validation Set	Test Set
Adenosis	444	284	71	89
Fibroadenoma	1014	649	162	203
Tubular Adenoma	453	290	72	91
Phyllodes Tumor	569	364	91	114
Ductal Carcinoma	3451	2209	552	690
Lobular Carcinoma	626	401	100	125
Mucinous Carcinoma	792	507	127	158
Papillary Carcinoma	560	358	90	112

### Data generation module

To enlarge the BACH dataset and enhance the model’s generalization ability, this study employed the StyleGAN2-ADA framework for image generation. Four pathological categories (Normal, Benign, InSitu, and Invasive) were used as conditional labels, and the model was trained in conditional generation mode. To ensure standardized image size and preserve pathological features, the original .tiff images were first converted to .png format for subsequent processing and storage. Each image was then centrally cropped to extract a 1536 × 1536-pixel block from the middle region, thereby retaining rich tissue structures while reducing background interference.

The cropped images were further resized to 1024 × 1024 resolution to match the input requirements of StyleGAN2-ADA, which improved both training efficiency and sample consistency while preserving key diagnostic information. A total of 3000 kimgs were used for model training, and ultimately 100 high-quality pathology images with 1024 × 1024 resolution were generated for each class. Fixed random seeds were applied during image generation to ensure reproducibility, while the class parameter explicitly specified the target category to achieve accurate conditional generation.

The generated images were subsequently employed for data augmentation and performance validation of the classification model.

### Loss function

In this study, we adopt the multi-class cross-entropy loss function, a widely used and well-established criterion in deep learning, in conjunction with our multimodal fusion neural network. This loss penalizes discrepancies between predicted distributions and ground-truth labels, thereby encouraging the model to learn discriminative deep feature representations for the complex and fine-grained task of pathology image classification. Within our framework, the multimodal fusion model integrates multi-level image features extracted from both the ResNet and ViT branches, while being trained end-to-end under a unified cross-entropy loss. This design enables the model to capture more accurate feature representations across different pathology types, particularly improving differentiation between subcategories with subtle or ambiguous boundaries (e.g., Benign vs. InSitu).The loss function $\:{\mathcal{L}}_{total}$ can be expressed as Eq. (1):

1 $$\:{\mathcal{L}}_{\varvec{t}\varvec{o}\varvec{t}\varvec{a}\varvec{l}}=\frac{1}{\varvec{N}}\sum\nolimits_{\varvec{i}=1}^{\varvec{N}}\mathcal{L}\left({\varvec{x}}^{\left(\varvec{i}\right)},{\varvec{y}}^{\left(\varvec{i}\right)}\right)=\frac{1}{\varvec{N}}\sum\nolimits_{\varvec{i}=1}^{\varvec{N}}\:\left(-\varvec{l}\varvec{o}\varvec{g}\:{\varvec{p}}_{{\varvec{y}}^{\left(\varvec{i}\right)}}^{\left(\varvec{i}\right)}\right)$$

where N denotes the number of samples in a batch; $\:{x}^{\left(i\right)}\:$ = [$\:{x}_{1}^{\left(i\right)},{x}_{2}^{\left(i\right)},\cdots,{x}_{C}^{\left(i\right)}$] is the un-normalized logit vector of the model output of the $\:i$-th sample; $\:{y}^{\left(i\right)}$ is the true category label of the $\:i$-th sample; $\:{p}_{{y}^{\left(i\right)}}^{\left(i\right)}$ is the predicted probability of the true category y in the $\:i$-th sample computed by the Softmax function.

Let the model output an unnormalized logit vector for one input image as shown in (2):

2 $$\:\varvec{x}=\left[{\varvec{x}}_{1},{\varvec{x}}_{2},\dots,{\varvec{x}}_{\varvec{C}}\right]\boldsymbol{\mathrm{\in}}\:{\varvec{R}}^{\varvec{C}}$$

Where C denotes the number of categories, which is 4 in the BACH classification task, corresponding to the four breast pathology states of Normal, Benign, InSitu and Invasive, respectively. In the BreakHis dataset, the number is 8, corresponding to eight categories (adenosis, fibroadenoma, phyllodes_tumor, tubular_adenoma, ductal_carcinoma, lobular_carcinoma, mucinous_carcinoma, papillary_carcinoma) of breast pathological conditions. In order to obtain the normalized probability distribution, the logit vector is first mapped to the predicted probability using the Softmax function as shown in (3):

3 $$\:{\varvec{p}}_{\varvec{i}}=\frac{{\varvec{e}}^{{\varvec{x}}_{\varvec{i}}}}{\sum\nolimits_{\varvec{j}=1}^{\varvec{C}}\:{\varvec{e}}^{{\varvec{x}}_{\varvec{j}}}},\:\:\varvec{i}=\boldsymbol{\mathrm{1}},\boldsymbol{\mathrm{2}},\boldsymbol{\mathrm{3}},\boldsymbol{\mathrm{4}}$$

Where $\:{p}_{i}$ denotes the probability that the model predicts the image to belong to class $\:i$. Let the true label of the image be y∈{1,2,3,4}, then the corresponding cross-entropy $\:{\mathcal{L}}_{total}(x,y)$-loss is defined as shown in (4):

4 $$\:{\mathcal{L}}_{\varvec{t}\varvec{o}\varvec{t}\varvec{a}\varvec{l}}(\varvec{x},\varvec{y})=-\varvec{l}\varvec{o}\varvec{g}\left({\varvec{p}}_{\varvec{y}}\right)=-{\varvec{x}}_{\varvec{y}}+\varvec{l}\varvec{o}\varvec{g}\left(\sum\nolimits_{\varvec{j}=1}^{\varvec{C}}\:{\varvec{e}}^{{\varvec{x}}_{\varvec{j}}}\right)$$

This loss function measures the distance between the model’s predictive distribution and the true labeling distribution. Its optimization goal is to maximize the prediction probability of the true categories to assign higher confidence to the true categories in the output of the classifier, so as to improve the discriminative ability of the model. During the training process, we use Mini-Batch gradient descent to iteratively update the model parameters, and in each round of iteration, the loss of the whole batch of samples is averaged, as shown in (5):

5 $$\:{\varvec{L}}_{\varvec{b}\varvec{a}\varvec{t}\varvec{c}\varvec{h}}=\frac{\boldsymbol{\mathrm{1}}}{\varvec{N}}\sum\nolimits_{\varvec{i}=\boldsymbol{\mathrm{1}}}^{\varvec{N}}\:\varvec{L}\left({\varvec{x}}^{\left(\varvec{i}\right)},{\varvec{y}}^{\left(\varvec{i}\right)}\right)$$

## Research methodology

**Fig. 1:**
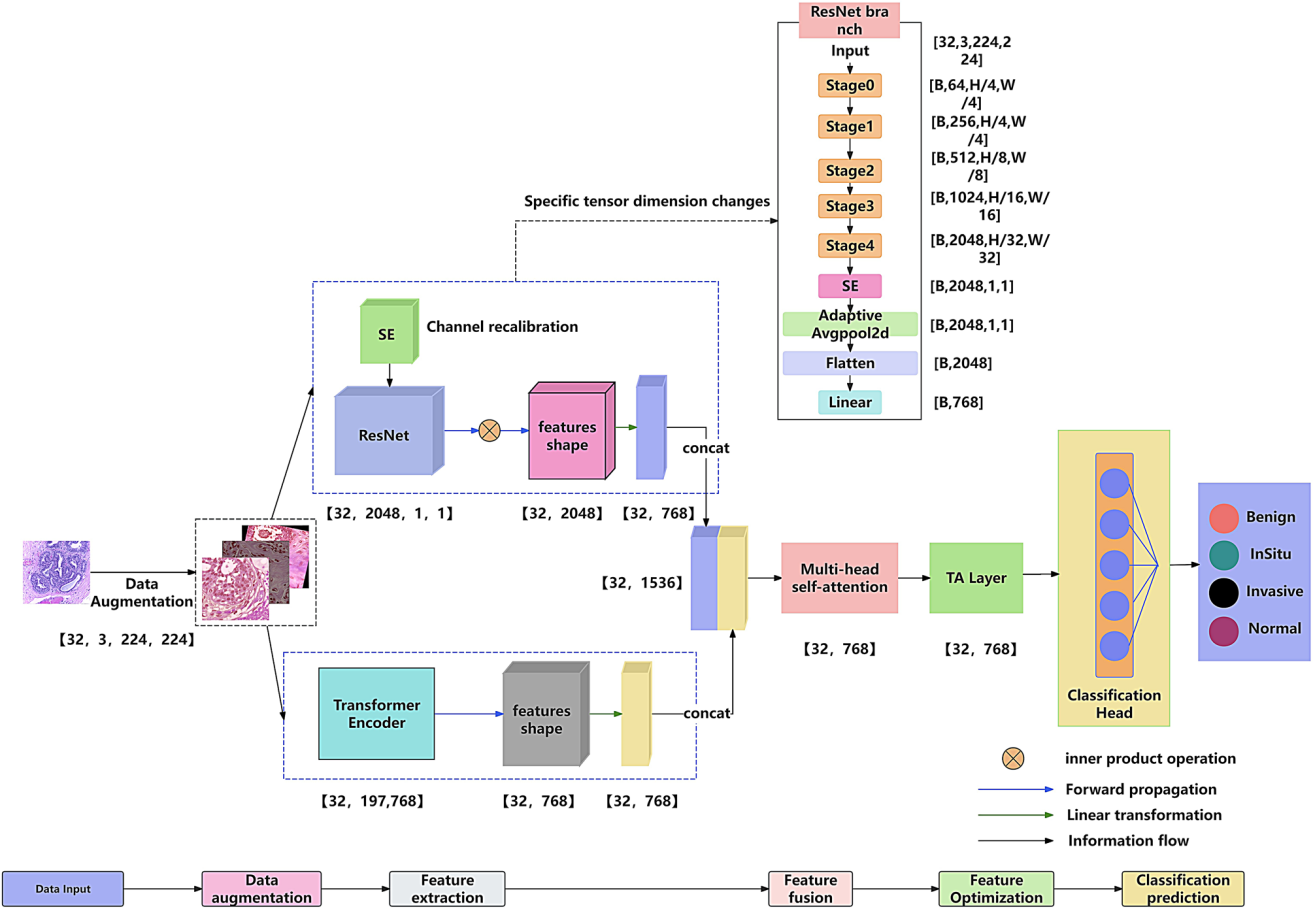
Model Architecture Diagram.The proposed model comprises a dual-branch structure integrating a ResNet50-based CNN and a Vision Transformer (ViT). The CNN branch captures fine-grained local texture features, while the ViT branch extracts hierarchical global semantic representations. The outputs are fused via a multimodal attention module and classified through a fully connected layer

In this paper, we propose ResViT-GANNet, a multimodal breast cancer pathology image classification network that integrates CNN and Transformer architectures. The overall model architecture is illustrated in Fig. 1. Specifically, the model employs ResNet50 and ViT as parallel feature extraction branches, incorporates a multimodal attention module for feature fusion, and ultimately performs classification and prediction.

Table 5 provides a detailed feature-by-feature comparison between ResViT-GANNet and other recent attention-based or hybrid models. It highlights critical architectural aspects, including the processing pipeline (parallel vs. serial), attention mechanisms (e.g., channel-only, spatial-only, multimodal), and fusion strategies. This side-by-side comparison clearly illustrates the architectural innovations of our parallel framework and the TAMA module, making their advantages more explicit and concrete.

**Table 5:** Architectural comparison of ResViT-GANNet with other attention and hybrid models

Feature /Component	ResViT-GANNet (Ours)	Typical Serial Hybrids	Standard ViT	CNNs with Attention (e.g., CBAM)
Processing Pipeline	Parallel (CNN &ViT concurrently)	Serial(CNN->Transformer)	N/A(ViT only)	N/A(CNN only)
Local Feature Extraction	ResNet-50	ResNet/DenseNet etc.	N/A(Patch projection)	Backbone CNN
Global Context Modeling	ViT(with multi-layer fusion)	Transformer Encoder	Transformer Encoder	Limited (receptive field)
Attention Mechanism	Multi-modal (TAMA) + Channel (SE)	Self-attention only	Self-attention only	Spatial and/or Channel
Fusion Strategy	TAMA	Concatenation/Simple projection	N/A	N/A
Handling Class Imbalance	StyleGAN2-ADA augmentation	Focal Loss/Weighted sampling	Focal Loss/Weighted sampling	Focal Loss/Weighted sampling
Interpretability	Grad-CAM visualization	Often not emphasized	Attention rollout	Grad-CAM

### Model execution steps

In the first stage, a dual-modal data enhancement strategy is employed. Spatial transformations (e.g., random cropping, rotation, and color jittering) are applied to the input images to improve the robustness of local features. Meanwhile, StyleGAN2-ADA is used to generate additional high-quality samples, thereby expanding the data distribution. The enhanced images are then fed into both the ResNet and Transformer branches to preserve key pathological information.

In the second stage, local features are extracted through the ResNet branch, where a squeeze-and-excitation (SE) module is introduced at the end of the convolutional layers to enhance channel attention. In parallel, the ViT branch captures global contextual information. [CLS] tokens from the 6th, 9th, and 12th layers are aggregated to form hierarchical global feature representations.

In the third stage, a multimodal attention mechanism is designed to fuse heterogeneous features. Specifically, features from the ResNet and ViT branches are concatenated along the channel dimension, projected into a unified feature space (768 dimensions), and processed by a multi-head self-attention mechanism to compute cross-modal correlation weights, thereby emphasizing complementary regions.

In the fourth stage, the fused feature vector is further refined using the Token-Aligned Attention (TA) layer, followed by average pooling over the sequence features.

In the fifth stage, the final fused representation is passed through the classification head, consisting of a fully connected layer and a dropout layer, which effectively suppresses overfitting. A softmax layer then outputs the class probabilities (e.g., four categories in the BACH dataset).

### Formal mathematical description

Let an input histopathology image be denoted as $\:X\in\:{\mathbb{R}}^{H\times\:W\times\:3}$. Our framework consists of a parallel CNN branch, a Transformer branch, and a Token-Aligned Multimodal Attention (TAMA) fusion module, followed by a classification head.

1. CNN branch with SE block

A ResNet-50 backbone extracts local feature maps:

6 $$\:{\varvec{F}}_{\varvec{c}\varvec{n}\varvec{n}}=\:{\varvec{f}}_{\varvec{R}\varvec{e}\varvec{s}\varvec{N}\varvec{e}\varvec{t}}(\varvec{X};{\varvec{\theta\:}}_{\varvec{c}\varvec{n}\varvec{n}})\boldsymbol{\mathrm{\in}}\:{\mathbb{R}}^{\varvec{B}\times\:\boldsymbol{\mathrm{2048}}\times\:\varvec{H}/\boldsymbol{\mathrm{32}}\times\:\varvec{W}/\boldsymbol{\mathrm{32}}}$$

The Squeeze-and-Excitation (*SE*) module performs channel recalibration:

7 $$\:\varvec{s}=\varvec{\sigma}\left({\varvec{W}}_{\boldsymbol{\mathrm{2}}}\:\varvec{\delta\:}\right({\varvec{W}}_{\boldsymbol{\mathrm{1}}}\cdot\:\varvec{G}\varvec{A}\varvec{P}\left({\varvec{F}}_{\varvec{c}\varvec{n}\varvec{n}}\right)\left)\right)$$

8 $$\:{\widetilde{\varvec{F}}}_{\varvec{c}\varvec{n}\varvec{n}}={\varvec{F}}_{\varvec{c}\varvec{n}\varvec{n}}\odot\varvec{s}$$

where GAP denotes global average pooling, δ is ReLU, σ is the sigmoid function, and ⊙ is channel-wise scaling. After pooling and projection:

9 $$\:{\varvec{z}}_{\varvec{c}\varvec{n}\varvec{n}}={\varvec{W}}_{\varvec{c}\varvec{n}\varvec{n}}\cdot\:\varvec{G}\varvec{A}\varvec{P}\left({\widetilde{\varvec{F}}}_{\varvec{c}\varvec{n}\varvec{n}}\right)\boldsymbol{\mathrm{\in}}\:{\mathbb{R}}^{\varvec{B}\times\:\varvec{d}}$$

(2) Transformer branch

The Vision Transformer encodes global contextual features. We extract CLS tokens from multiple layers:

10 $$\:{\varvec{z}}_{\varvec{v}\varvec{i}\varvec{t}}=\frac{\boldsymbol{\mathrm{1}}}{\boldsymbol{\mathrm{3}}}\sum\limits_{\varvec{l}\boldsymbol{\mathrm{\in}}\:\{\boldsymbol{\mathrm{6,9,12}}\}}{\varvec{h}}_{\varvec{l}}^{\left[\varvec{C}\varvec{L}\varvec{S}\right]}\boldsymbol{\mathrm{\in}}\:{\mathbb{R}}^{\varvec{B}\times\:\varvec{d}}$$

where $\:{h}_{l}^{\left[CLS\right]}$ is the [*CLS*] embedding from the *l*-th Transformer block.

(3) Token-aligned multimodal attention (TAMA)

The CNN and ViT tokens are concatenated:

11 $$\:\varvec{z}={[\varvec{z}}_{\mathbf{c}\mathbf{n}\mathbf{n}};{\varvec{z}}_{\mathbf{v}\mathbf{i}\mathbf{t}}]{\mathbf{W}}_{\mathbf{f}}\:\boldsymbol{\mathrm{\in}}\:{\mathbb{R}}^{\boldsymbol{\mathrm{B}}\times\:\boldsymbol{\mathrm{d}}}$$

A multi-head self-attention mechanism is applied:

12 $$\:\varvec{Q},\varvec{K},\varvec{V}=\varvec{z}{\varvec{W}}_{\varvec{Q}},\varvec{z}{\varvec{W}}_{\varvec{K}},\varvec{z}{\varvec{W}}_{\varvec{V}}$$

13 $$\:\varvec{A}=\varvec{S}\varvec{o}\varvec{f}\varvec{t}\varvec{m}\varvec{a}\varvec{x}\left(\frac{\varvec{Q}{\varvec{K}}^{\varvec{T}}}{\sqrt{\varvec{d}}}\right)\varvec{V}$$

The attended representation is further refined by a Token-Aligned layer, and average pooling produces the fused feature:

14 $$\:{\varvec{z}}_{\varvec{f}\varvec{u}\varvec{s}\varvec{e}\varvec{d}}=\varvec{M}\varvec{e}\varvec{a}\varvec{n}\varvec{P}\varvec{o}\varvec{o}\varvec{l}\left(\varvec{A}\right)$$

(4) Classification head

Finally, the fused feature is passed through a feedforward classifier:

15 $$\:\widehat{\varvec{y}}=\varvec{S}\varvec{o}\varvec{f}\varvec{t}\varvec{m}\varvec{a}\varvec{x}\left({\varvec{W}}_{\varvec{c}}\cdot\:\varvec{\phi\:}\left({\varvec{z}}_{\varvec{f}\varvec{u}\varvec{s}\varvec{e}\varvec{d}}\right)+{\varvec{b}}_{\varvec{c}}\right)$$

where $\varvec{\phi}$ denotes ReLU activation with dropout regularization.

### ResNet branch with pathology-specific channel recalibration

The ResNet branch is designed to extract hierarchical local features from histopathological images. It follows a five-stage architecture (Stage 0–4), where the spatial resolution is progressively reduced while the channel dimension increases. Stage 0 consists of a 7 × 7 convolution followed by 3 × 3 max pooling, reducing the input from 224 × 224 to 56 × 56. Stages 1–4 are composed of bottleneck-based residual blocks, capturing features at increasing abstraction levels, from low-level textures to high-level semantic structures.

A key innovation of our ResNet branch lies in the integration of a pathology-specific channel recalibration mechanism. After Stage 4, we introduce the Squeeze-and-Excitation (SE) module, which first applies global average pooling to generate a compact descriptor, then passes it through a two-layer fully connected network to learn channel-wise weights. These weights recalibrate the feature maps via element-wise multiplication, effectively emphasizing pathology-relevant channels (e.g., regions with high nuclear density, chromatin irregularity, or abnormal glandular structures) while suppressing irrelevant background signals such as fat and empty spaces. This directly addresses a major challenge in histopathological image analysis: a large proportion of tissue regions contain diagnostically irrelevant background, which distracts conventional attention modules and dilutes lesion-specific features.

**Fig. 2:**
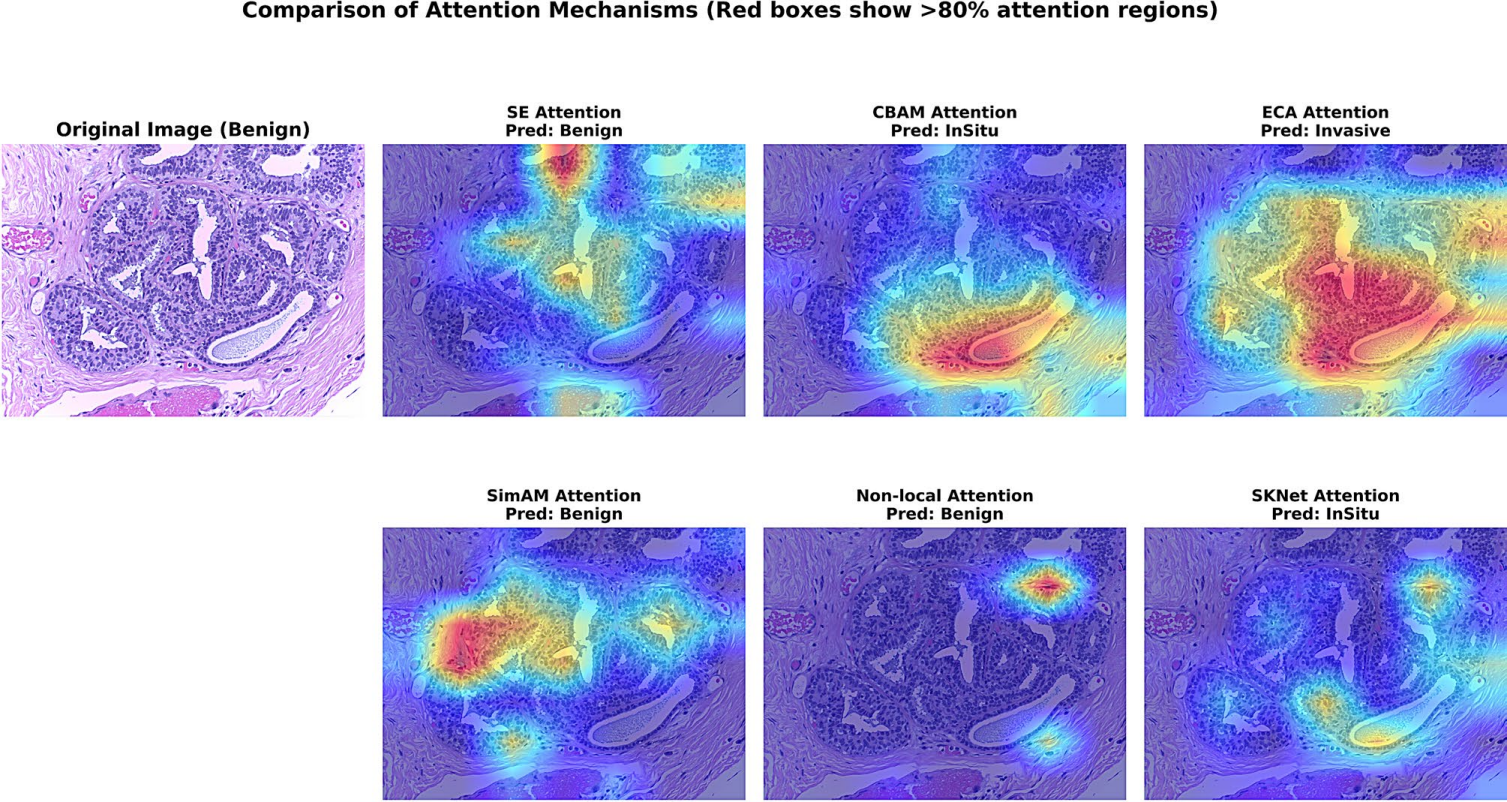
The image shows the heatmap and prediction results of different attention mechanisms

Unlike generic attention mechanisms developed for natural images (e.g., CBAM, SimAM, ECA, Non-local, SKNet), which often fail to suppress such interference, our systematic comparison (Table 6) shows that SE consistently outperforms alternatives in both accuracy and stability. In particular, SE’s ability to recalibrate channels according to the global pathology context aligns more closely with the diagnostic reasoning of pathologists, who naturally prioritize cellular-level discriminative features (e.g., nuclear morphology, chromatin texture) over irrelevant global structures. As further illustrated in Fig. 2, SE attention sharply highlights tumor-relevant regions, whereas other mechanisms either spread attention too broadly (Non-local), misallocate focus to structured but nondiscriminative tissues (CBAM, SKNet), or produce unstable patterns across samples (SimAM).

Finally, the recalibrated features are aggregated by adaptive average pooling to produce a global descriptor, which is projected into a 768-dimensional embedding space to align with the Transformer branch. Collectively, the ResNet branch introduces two major innovations: (1) a hierarchical residual design tailored for multi-scale local pathology feature extraction, and (2) a pathology-optimized channel recalibration mechanism that enhances discriminative power, improves generalization, and provides empirical guidance for attention selection in histopathological image analysis. These innovations go beyond simple architectural adaptation, representing pathology-specific optimizations that significantly improve both model performance and interpretability.

**Table 6:** Comparison of different attention mechanisms on pathological image classification

Module	Type	BACH (F1-score %)	BreakHis (F1- score %)	Key Findings in our Experiments
SE	Channel Attention	96.35	98.23	Achieved the best performance; effectively emphasized tumor-related channels (e.g., high nuclear density, nuclear atypia), significantly improved classification accuracy, without introducing obvious overfitting.
CBAM	Channel + Spatial Attention	93.81	96.80	Susceptible to irrelevant regions in complex backgrounds; effective in some structured tissues but overall performance unstable.
ECA	Channel Attention	91.50	95.20	Limited cross-channel interaction; weaker in capturing fine-grained nuclear morphology compared to SE.
SimAM	Parameter-free Attention	93.20	97.10	Unstable performance; results varied across datasets and hyperparameters, with relatively poor robustness.
Non-local	Global Self-Attention	94.90	97.50	Very high computational cost, making it less feasible for high-resolution pathological images.
SKNet	Dynamic Kernel Attention	94.10	96.90	Provides improvements for multi-scale features (cells, glands), but computationally expensive.

### Transformer branch

**Fig. 3:**
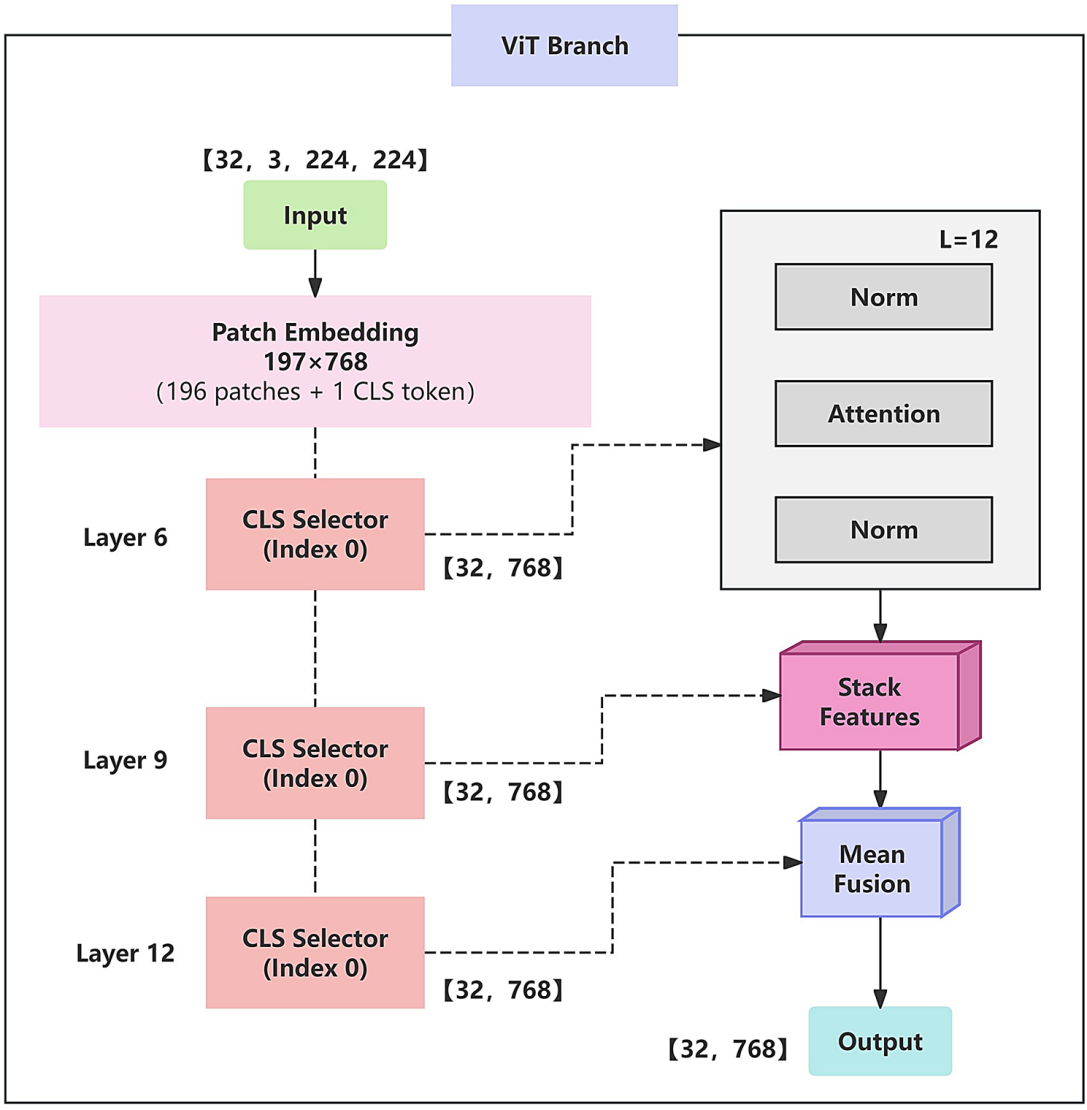
ViT branch structure diagram.The architecture integrates patch tokens and [CLS] tokens from the 6th, 9th, and 12th Transformer layers to capture multilevel semantic information, enhancing the global context representation of histopathological features

As shown in Fig. 3, the ViT branch enhances the standard Vision Transformer by introducing hierarchical feature extraction and fusion mechanisms, aiming to better capture multi-scale semantic information for complex tasks such as breast cancer histopathological image analysis.

Specifically, the input image $\:I\in\:{\mathbb{R}}^{H\times\:W\times\:3}$ is first divided into N non-overlapping patches of size $\:P\times\:P$. Each patch is flattened and linearly projected into a token vector of fixed dimension:

16 $$\:{\varvec{x}}_{\varvec{i}}=\varvec{L}\varvec{i}\varvec{n}\varvec{e}\varvec{a}\varvec{r}\left(\varvec{F}\varvec{l}\varvec{a}\varvec{t}\varvec{t}\varvec{e}\varvec{n}\left({\varvec{p}}_{\varvec{i}}\right)\right)\boldsymbol{\mathrm{\in}}\:{\mathbb{R}}^{\varvec{D}},\quad\:\varvec{i}=\boldsymbol{\mathrm{1,2,}}\dots\:,\varvec{N}$$

These patch tokens, along with a learnable classification token [CLS], are concatenated to form the input sequence:

17 $$\:\varvec{X}=\left[{\varvec{x}}_{\varvec{C}\varvec{L}\varvec{S}},{\varvec{x}}_{\boldsymbol{\mathrm{1}}},{\varvec{x}}_{\boldsymbol{\mathrm{2}}},\dots,{\varvec{x}}_{\varvec{N}}\right]+\varvec{P}\varvec{E}$$

Where PE represents position encoding. The resulting input $\:X\in\:{\mathbb{R}}^{(N+1)\times\:D}$ is then fed into a stack of 12 Transformer encoder layers. Each layer consists of a Layer Normalization, MSA, and MLP. The MSA operation is formulated as:

18 $$\:\varvec{M}\varvec{S}\varvec{A}\left(\varvec{Q},\varvec{K},\varvec{V}\right)=\varvec{C}\varvec{o}\varvec{n}\varvec{c}\varvec{a}\varvec{t}\left({\varvec{h}\varvec{e}\varvec{a}\varvec{d}}_{\boldsymbol{\mathrm{1}}},\dots,{\varvec{h}\varvec{e}\varvec{a}\varvec{d}}_{\varvec{h}}\right){\varvec{W}}^{\varvec{O}}$$

In the standard ViT architecture, the output of the [CLS] token from the final (12th) layer is typically used as the image-level representation:

19 $$\:\varvec{z}={\varvec{x}}_{\varvec{c}\varvec{l}\varvec{s}}^{\left(\boldsymbol{\mathrm{12}}\right)}\boldsymbol{\mathrm{\in}}\:{\mathbb{R}}^{\varvec{D}}$$

In contrast, we propose a hierarchical feature fusion strategy that extracts [CLS] tokens from the 6th, 9th, and 12th layers:

20 $$\:{\varvec{z}}_{\boldsymbol{\mathrm{6}}}={\varvec{x}}_{\varvec{c}\varvec{\boldsymbol{\mathrm{l}}}\varvec{s}}^{\left(\boldsymbol{\mathrm{6}}\right)},\quad{\varvec{z}}_{\boldsymbol{\mathrm{9}}}={\varvec{x}}_{\varvec{c}\varvec{l}\varvec{s}}^{\left(\boldsymbol{\mathrm{9}}\right)},\quad{\varvec{z}}_{\boldsymbol{\mathrm{12}}}={\varvec{x}}_{\varvec{c}\varvec{l}\varvec{s}}^{\left(\boldsymbol{\mathrm{12}}\right)}\boldsymbol{\mathrm{\in}}\:{\mathbb{R}}^{\varvec{D}}$$

These representations are then aggregated via averaging to obtain the final ViT output:

21 $$\:{\varvec{z}}_{\varvec{v}\varvec{i}\varvec{t}}=\frac{\boldsymbol{\mathrm{1}}}{\boldsymbol{\mathrm{3}}}\left({\varvec{z}}_{\boldsymbol{\mathrm{6}}}+{\varvec{z}}_{\boldsymbol{\mathrm{9}}}+{\varvec{z}}_{\boldsymbol{\mathrm{12}}}\right)\boldsymbol{\mathrm{\in}}\:{\mathbb{R}}^{\varvec{D}}$$

This hierarchical fusion strategy allows the model to combine fine-grained details with high-level semantics, enhancing its ability to capture complex tissue structures and subtle morphological variations.

### Multimodal feature fusion module

**Fig. 4:**
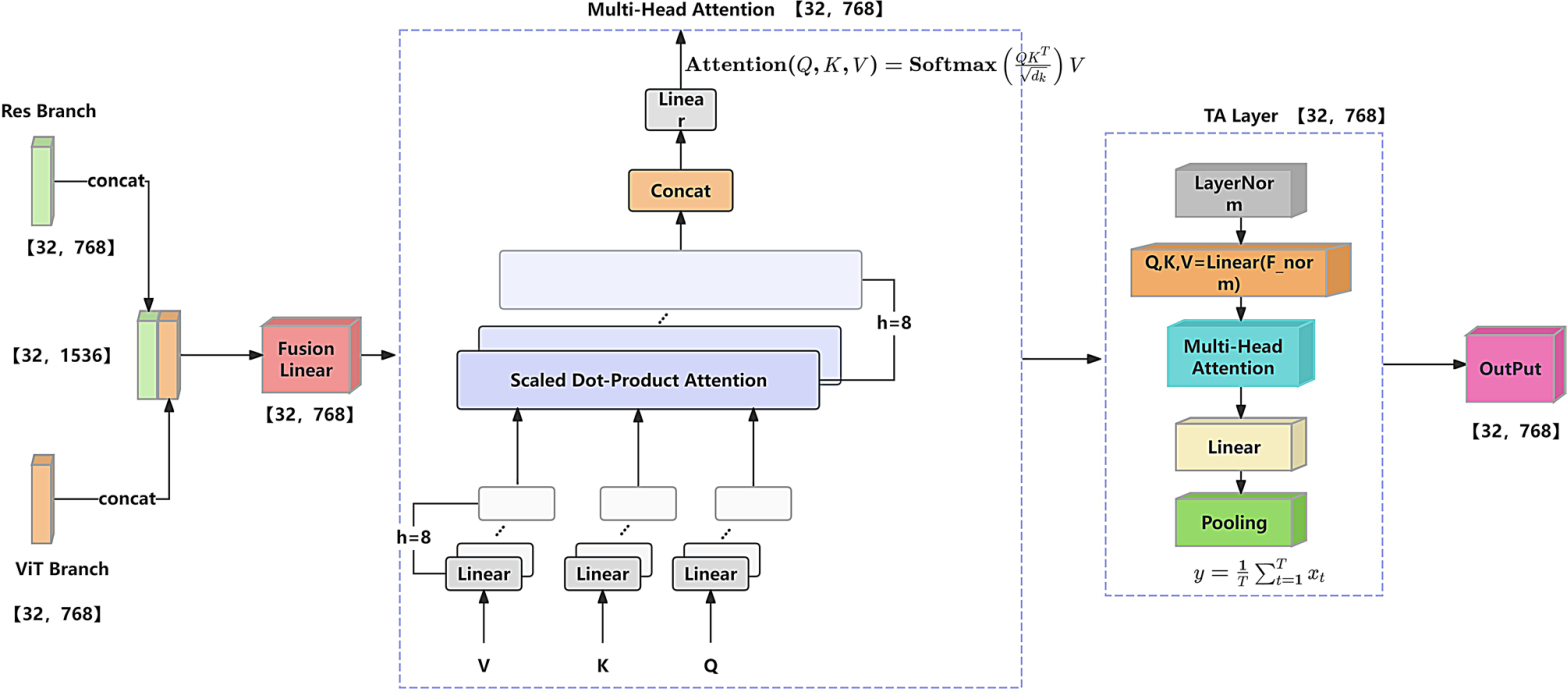
Multimodal feature fusion module.Feature maps from the CNN and ViT branches are concatenated and fed into a two-stage fusion unit, composed of a Multi-Head Self-Attention (MHSA) mechanism followed by a Token Alignment (TA) module, enabling effective cross-modal feature interaction

This paper introduces a novel Token-Aligned Multimodal Attention (TAMA) Module to effectively fuse spatial features from ResNet and semantic features from ViT. The module adopts a two-stage attention mechanism to enhance cross-modal complementarity and contextual understanding, as illustrated in Fig. 4.

First, features from the ResNet branch $\:{F}_{ResNet}$ $\in$ ${\mathbb{R}}^{B\times\:d}$ and the ViT branch $\:{F}_{ViT}$ $\in$ ${\mathbb{R}}^{B\times\:d}$ are concatenated along the channel dimension to form an initial fused representation:

22 $$\:{\varvec{F}}_{\varvec{f}\varvec{u}\varvec{s}\varvec{e}\varvec{d}}=\varvec{C}\varvec{o}\varvec{n}\varvec{c}\varvec{a}\varvec{t}\left({\varvec{F}}_{\varvec{R}\varvec{e}\varvec{s}\varvec{N}\varvec{e}\varvec{t}},\quad{\varvec{F}}_{\varvec{V}\varvec{i}\varvec{T}}\right)\boldsymbol{\mathrm{\in}}\:{\mathbb{R}}^{\varvec{B}\times\:\boldsymbol{\mathrm{2}}\varvec{d}}$$

To maintain consistent dimensionality and avoid parameter explosion, a linear projection reduces the fused features to d = 768:

23 $$\:{\varvec{F}}_{\varvec{r}\varvec{e}\varvec{d}\varvec{u}\varvec{c}\varvec{e}\varvec{d}}=\varvec{W}\cdot\:{\varvec{F}}_{\varvec{f}\varvec{u}\varvec{s}\varvec{e}\varvec{d}}+\varvec{b}$$

This dimensionality alignment retains complementary cross-modal information more effectively than simple concatenation or averaging, which often discard discriminative details.

In the first attention stage, a Multi-Head Self-Attention (MHSA) mechanism is used to model localized dependencies:

24 $$\begin{aligned}&\:\varvec{A}\varvec{t}\varvec{t}\varvec{e}\varvec{n}\varvec{t}\varvec{i}\varvec{o}\varvec{n}\left(\varvec{Q},\varvec{K},\varvec{V}\right)=\varvec{S}\varvec{o}\varvec{f}\varvec{t}\varvec{m}\varvec{a}\varvec{x}\left(\frac{\varvec{Q}{\varvec{K}}^{\varvec{T}}}{\sqrt{{\varvec{d}}_{\varvec{k}}}}\right)\varvec{V},\cr&\quad\varvec{Q}=\varvec{K}=\varvec{V}={\varvec{F}}_{\varvec{r}\varvec{e}\varvec{d}\varvec{u}\varvec{c}\varvec{e}\varvec{d}}\end{aligned}$$

This module uses 8 attention heads, each attending to different subspaces to enrich the representational diversity and expand the receptive field.In the second stage, a custom TA Layer is introduced to model long-range dependencies and global contextual relationships. It consists of four sequential steps:

Layer normalization is applied to stabilize feature distributions and improve cross-modal alignment:

25 $$\:{\varvec{F}}_{\varvec{n}\varvec{o}\varvec{r}\varvec{m}}=\varvec{L}\varvec{a}\varvec{y}\varvec{e}\varvec{r}\varvec{N}\varvec{o}\varvec{r}\varvec{m}\left(\varvec{X}\right)$$

Query (Q), Key (K), and Value (V) matrices are generated via shared linear projections:

26 $$\:\varvec{Q},\varvec{K},\varvec{V}=\varvec{L}\varvec{i}\varvec{n}\varvec{e}\varvec{a}\varvec{r}\left({\varvec{F}}_{\varvec{n}\varvec{o}\varvec{r}\varvec{m}}\right)$$

Multi-head attention is computed across h heads:

27 $$\:\varvec{M}\varvec{H}\varvec{A}\left(\varvec{Q},\varvec{K},\varvec{V}\right)=\sum\limits_{\varvec{i}=\boldsymbol{\mathrm{1}}}^{\varvec{h}}{\varvec{A}\varvec{t}\varvec{t}\varvec{e}\varvec{n}\varvec{t}\varvec{i}\varvec{o}\varvec{n}}_{\varvec{i}}\left({\varvec{Q}}_{\varvec{i}},{\varvec{K}}_{\varvec{i}},{\varvec{V}}_{\varvec{i}}\right)$$

The multi-head outputs are concatenated and projected back to the original dimension:

28 $$\:\widehat{\varvec{X}}=\varvec{L}\varvec{i}\varvec{n}\varvec{e}\varvec{a}\varvec{r}\left(\varvec{M}\varvec{H}\varvec{A}\left(\varvec{Q},\varvec{K},\varvec{V}\right)\right)$$

Finally, the sequence output from the TA Layer is aggregated into a global representation vector using average pooling:

29 $$\:\varvec{y}=\frac{\boldsymbol{\mathrm{1}}}{\varvec{T}}\sum\limits_{\varvec{t}=\boldsymbol{\mathrm{1}}}^{\varvec{T}}{\varvec{x}}_{\varvec{t}}$$

This average pooling operation is both efficient and stable, allowing the model to retain essential semantic structures without added complexity—an advantage over traditional attention-based fusion methods.

Experimental results on the BACH and BreakHis datasets demonstrate that the proposed multimodal attention module consistently outperforms conventional fusion strategies by effectively integrating heterogeneous features with minimal computational cost. This leads to improved classification accuracy and enhanced model robustness in pathological image analysis.

### Model visualization and analysis module

In order to enhance the interpretability of this model in the task of breast cancer pathology image classification, enabling its classification results to provide guidance to pathologists for diagnosis, Grad-CAM [26] is introduced in this paper to visualize the regions of interest of the model, the generation of category-related spatial attention heat maps effectively reveals the discriminative regions on which the model relies in the categorization decision process. In this study, we select three representative convolutional layers of ResNet (the last layer of the first layer3 Block, the 2nd Block and the last Block in layer4), extract their forward feature maps and compute the gradient information of the predicted categories respectively, further generate the class activation maps (CAMs) of each layer, finally fusing them to increase the stability of the heat map and semantic expressiveness. The overall process is described as follows.

Suppose the input image is X, the model predicts category c.We extract the forward feature maps from the three target convolutional layers of the ResNet branch(denoted as $\:{l}_{1}$, $\:{l}_{2}$, $\:{l}_{3}$) extract its forward feature map $\:{A}^{\left(k\right)}\in\:{\mathbb{R}}^{{C}_{k}\times\:{H}_{k}\times\:{W}_{k}}$,the gradient of category c is obtained by backpropagation $\:\frac{\partial\:{y}^{c}}{\partial\:{A}^{\left(k\right)}}$.For each target layer$\:{\:l}_{k}$, the gradients are aggregated along the spatial dimension by global average pooling to obtain the weight of the $\:i$-th channel $\alpha_{i}^{(k)}$, the formula is as shown in (30):

30 $$\:{\varvec{\alpha}}_{\varvec{i}}^{\left(\varvec{k}\right)}=\frac{\boldsymbol{\mathrm{1}}}{{\varvec{H}}_{\varvec{k}}{\varvec{W}}_{\varvec{k}}}\sum\limits_{\varvec{h}=\boldsymbol{\mathrm{1}}}^{{\varvec{H}}_{\varvec{k}}}\sum\limits_{\varvec{w}=\boldsymbol{\mathrm{1}}}^{{\varvec{W}}_{\varvec{k}}}\frac{\partial{\varvec{y}}^{\varvec{c}}}{\partial{\varvec{A}}_{\varvec{i},\varvec{h},\varvec{w}}^{\left(\varvec{k}\right)}}$$

Among them, $\:{A}_{i,h,w}^{\left(k\right)}$ represents the activation value of the position ($\:h,w$) on the feature map of the $\:i$-th channel; $\:{y}^{c}$ represents the output score of category $\:c$.

The feature map of each channel is weighted and summed with the corresponding weight to obtain the heat map $\:{L}_{c}^{\left(k\right)}$ corresponding to the category $\:c$ as shown in (31):

31 $$\:{\varvec{L}}_{\varvec{c}}^{\left(\varvec{k}\right)}=\varvec{R}\varvec{e}\varvec{L}\varvec{U}\left(\sum\limits_{\varvec{i}}{\varvec{\alpha\:}}_{\varvec{i}}^{\left(\varvec{k}\right)}\cdot{\varvec{A}}_{\varvec{i}}^{\left(\varvec{k}\right)}\right)$$

In order to promote the robustness and spatial representation of the heatmap, we post-dimensionally average the three layers of output CAMs, which is finally fused to the heat map $\:{L}_{c}^{fusion}$ is shown in (32):

32 $$\:{\varvec{L}}_{\varvec{c}}^{\varvec{f}\varvec{u}\varvec{s}\varvec{i}\varvec{o}\varvec{n}}=\frac{\boldsymbol{\mathrm{1}}}{\boldsymbol{\mathrm{3}}}\sum\limits_{\varvec{k}=\boldsymbol{\mathrm{1}}}^{\boldsymbol{\mathrm{3}}}\varvec{R}\varvec{e}\varvec{s}\varvec{i}\varvec{z}\varvec{e}\left({\varvec{L}}_{\varvec{c}}^{\left(\varvec{k}\right)}\right)$$

Among them, Resize denotes the up-sampling of each CAM to unify to the same resolution.

Ultimately, the normalized $\:{L}_{c}^{fusion}$ is mapped to a pseudo-color heatmap and fused to the original image for display, which is used to visualize the discriminative region of interest to the model, the formula is shown in (33):

33 $$\:\varvec{O}\varvec{v}\varvec{e}\varvec{r}\varvec{l}\varvec{a}\varvec{y}=\varvec{I}\varvec{m}\varvec{a}\varvec{g}\varvec{e}\cdot\:(\boldsymbol{\mathrm{1}}-\varvec{\alpha\:})+\varvec{H}\varvec{e}\varvec{a}\varvec{t}\varvec{m}\varvec{a}\varvec{p}\cdot\:\varvec{\alpha\:}$$

### Statistical analysis

To evaluate the statistical significance and practical relevance of performance differences between ResViT-GANNet and competing baseline models, we conducted pairwise paired *t*-tests and calculated Cohen’s *d* effect sizes for each comparison. The *p*-value from the *t*-test indicates whether the observed differences are statistically significant, with thresholds of *p* < 0.05 considered significant and *p* < 0.01 highly significant. Cohen’s *d* was used to quantify the effect size, reflecting the magnitude of difference between models, and interpreted according to standard guidelines:

1. Small effect: *d* = 0.2.
2. Medium effect: *d* = 0.5.
3. Large effect: *d* ≥ 0.8.

To ensure robustness and mitigate the impact of random variability during training, each model was independently trained and evaluated across ten repeated runs with different random seeds, using consistent dataset splits.

Most performance improvements achieved by ResViT-GANNet were associated with *p*-values < 0.01 and large effect sizes (*d* > 1.0), indicating both statistical and practical superiority over baseline methods. Statistical analysis over these ten runs confirmed highly significant improvements (*p* < 0.01), with all pairwise comparisons yielding large effect sizes (*d* > 1.0), further supporting the practical relevance of the results.

All statistical analyses were performed in Python using the SciPy and Statsmodels libraries. Where applicable, Bonferroni correction was applied to adjust for multiple comparisons and reduce the risk of Type I errors.

## Results

### Evaluation indicators

In order to comprehensively evaluate the performance of the proposed model in the task of breast cancer pathology image classification, this study uses a variety of commonly used classification metrics for quantitative analysis, including classification accuracy, precision, recall, and F1 score.

1. Classification accuracy

Classification accuracy, the most commonly used classification performance metric, measures the percentage of correct predictions made by the model overall. Accuracy is intuitive and easy to interpret, allowing for a quick snapshot of the model’s performance on the entire test set. Equation (34) is calculated as shown below:

34 $$\:\varvec{A}\varvec{c}\varvec{c}\varvec{u}\varvec{r}\varvec{a}\varvec{c}\varvec{y}\:=\:\frac{\varvec{T}\varvec{P}+\varvec{T}\varvec{N}}{\varvec{T}\varvec{P}+\varvec{T}\varvec{N}+\varvec{F}\varvec{P}+\varvec{F}\varvec{N}}$$

where TP, TN, FP, and FN denote the true, true-negative, false-positive, and false-negative classes, respectively.

(2) Precision

Precision indicates the proportion of samples predicted by the model to belong to a particular category that actually belong to that category, and it measures the model’s ability to suppress false positives. The formula (35) is shown below:

35 $$\:\varvec{P}\varvec{r}\varvec{e}\varvec{c}\varvec{i}\varvec{s}\varvec{i}\varvec{o}\varvec{n}\:=\:\frac{\varvec{T}\varvec{P}}{\varvec{T}\varvec{P}+\varvec{F}\varvec{P}}$$

(3) Recall

Recall represents the proportion of positive class samples successfully identified by the model, high recall means that the model can capture more cancer cases. In the case of unbalanced datasets, it can effectively assess the performance of the model in a few classes. This metric reflects the model’s tolerance of false negatives, calculated by the formula (36) shown below:

36 $$\:\varvec{R}\varvec{e}\varvec{c}\varvec{a}\varvec{l}\varvec{l}\:=\:\frac{\varvec{T}\varvec{P}}{\varvec{T}\varvec{P}+\varvec{F}\varvec{N}}$$

(4) F1 score

The F1 Score is a reconciled average of Precision and Recall, which combines the two metrics and is used to weigh the relationship between them. It is particularly effective when targeting category imbalance, providing a fairer reflection of the model’s performance across categories, calculated as shown in Eq. (37) below:

37 $$\:\varvec{F}\boldsymbol{\mathrm{1}}\:=\:\frac{\boldsymbol{\mathrm{2}}\times\:\varvec{P}\varvec{r}\varvec{e}\varvec{c}\varvec{i}\varvec{s}\varvec{i}\varvec{o}\varvec{n}\times\:\varvec{R}\varvec{e}\varvec{c}\varvec{a}\varvec{l}\varvec{l}}{\varvec{P}\varvec{r}\varvec{e}\varvec{c}\varvec{i}\varvec{s}\varvec{i}\varvec{o}\varvec{n}+\varvec{R}\varvec{e}\varvec{c}\varvec{a}\varvec{l}\varvec{l}}$$

### Implementation details

This study is based on the PyTorch deep learning framework, all experiments are conducted on a server equipped with NVIDIA RTX 4090 GPU (24GB of video memory), Intel Xeon(R) Gold 6430 CPU, Ubuntu 22.04 OS, Python version 3.12. The experimental environment is configured as follows: PyTorch version 2.5.1, CUDA driver version 560.35.03, CUDA runtime version 12.4, CUDA toolkit version 12.6.

We trained the model using the AdamW optimizer, with an initial learning rate of 2 × 10⁻⁴, a weight decay coefficient of 0.01, and a total of 50 epochs with a batch size of 32. To improve training efficiency and reduce GPU memory consumption, Automatic Mixed Precision (AMP) training was employed. The learning rate was scheduled using Cosine Annealing with Warmup, which alleviates instability during the early training phase. To prevent overfitting, we incorporated an early stopping mechanism, terminating training if the validation loss failed to improve for 15 consecutive epochs.

Throughout training, we recorded and visualized the accuracy and loss curves for both training and validation sets, and ultimately preserved the model checkpoint with the best validation performance. This experimental setup demonstrated strong stability and generalization capability for breast cancer histopathology image classification, while also providing a reliable hardware and software foundation for subsequent research on multi-view fusion models.

### Classification results

#### Classification results based on BACH dataset

With the high resolution of the BACH dataset and the richness of pathology information, the model proposed in this study achieved excellent classification performance, the overall classification accuracy of the model on the test set reached 96.403%, the AUC value reached 99.538%, which showed strong discriminative ability and stability. Table 7 below demonstrates the specific values of each classification result.

**Table 7:** Classification results of BACH dataset

Class	Acc (%)	Prec (%)	Rec (%)	F1 (%)
Benign	95.181	94.611	95.181	94.895
InSitu	94.888	96.743	94.888	95.806
Invasive	98.837	99.415	98.837	99.125
Normal	96.552	94.595	96.552	95.563

Class, Four different breast cancer categories in the BACH dataset; ACC, Accuracy indicates the overall proportion of correct predictions; Prec, Precision refers to the proportion of predicted positive samples that are truly positive, reflecting the model’s ability to reduce false positives; Rec, Recall represents the proportion of actual positives correctly identified, indicating sensitivity and the model’s ability to reduce false negatives—especially important for imbalanced datasets; F1,F1 score is the harmonic mean of precision and recall, providing a balanced evaluation in the presence of class imbalance.

**Fig. 5:**
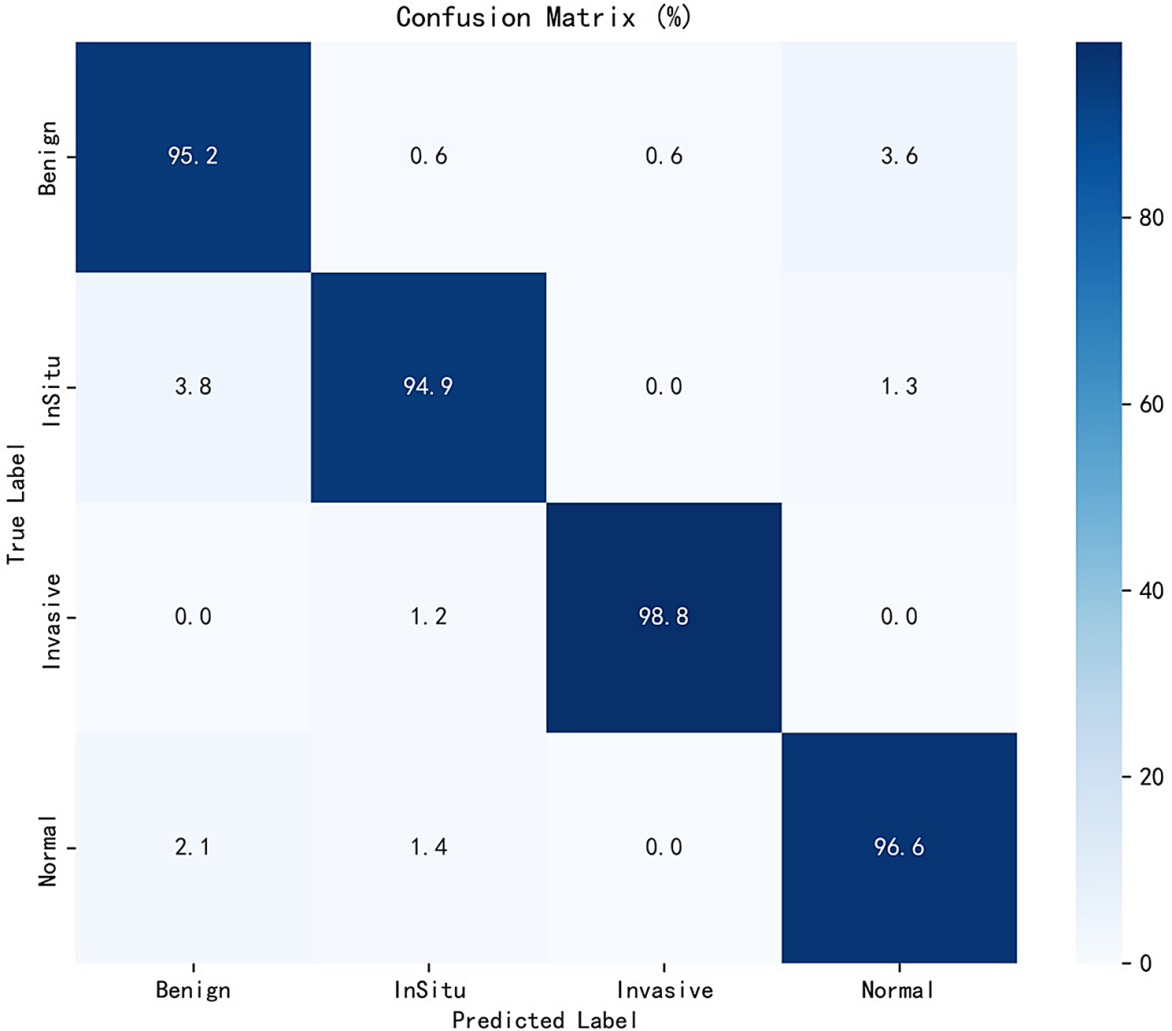
BACH-Confusion. The proposed model achieves high classification accuracy across four histopathological categories, with minimal confusion between morphologically similar classes

Figure 5 presents the confusion matrix on the BACH dataset, illustrating the model’s ability to classify four types of breast cancer pathology images. Most misclassifications occur among Benign, Normal, and InSitu classes, likely due to their morphological similarities and subtle boundaries.

**Fig. 6:**
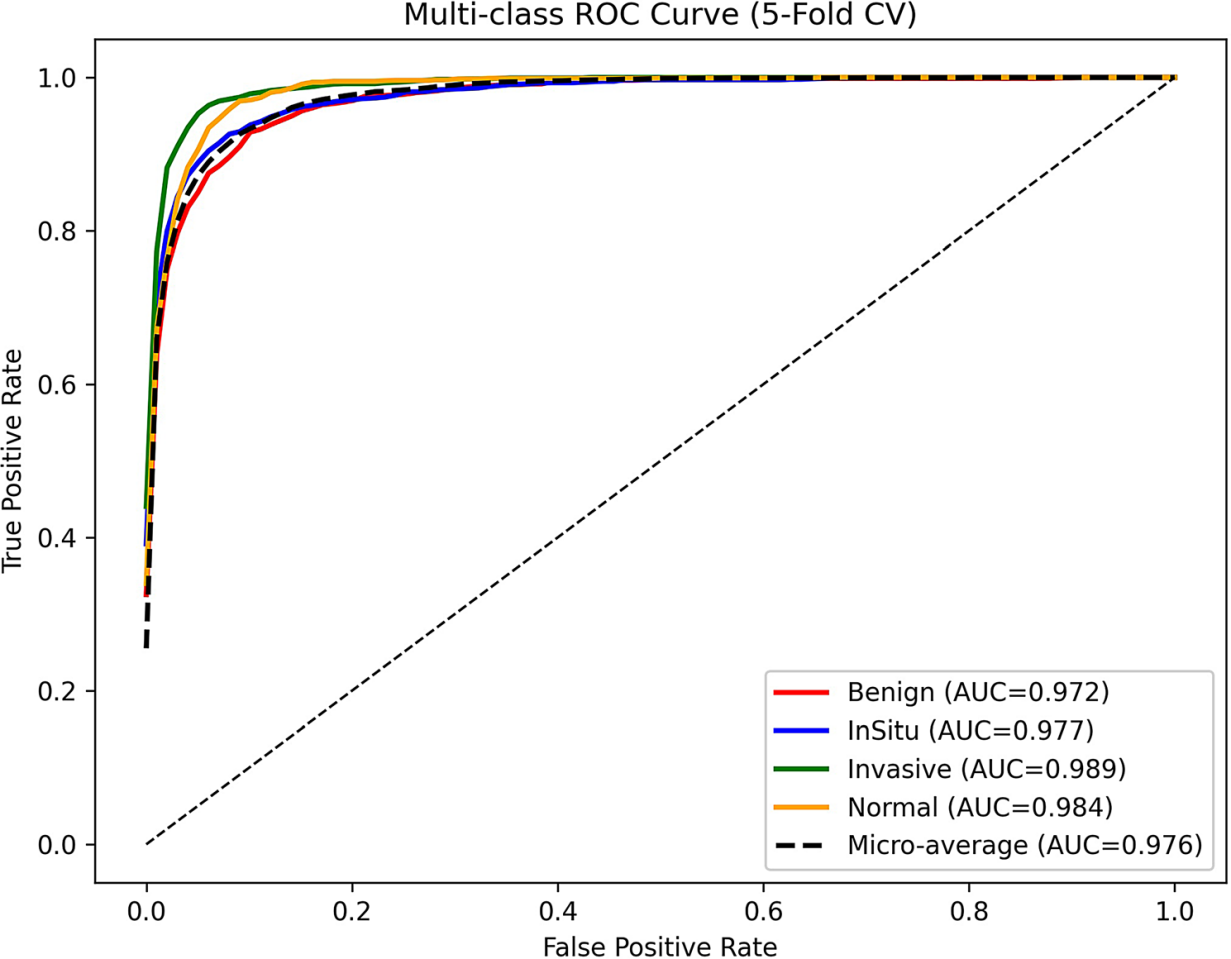
Multi-class ROC curve. Multi-class ROC curves of ResViT-GANNet obtained using 5-fold cross-validation. Each curve represents one class (Benign, InSitu, Invasive, Normal) with the corresponding AUC values, while the micro-average AUC reflects the overall performance

Figure 6. Multi-class ROC curves generated using 5-fold cross-validation. Each curve corresponds to one of the four classes—Benign, InSitu, Invasive, and Normal—with AUC values shown in the legend. The micro-average AUC is also reported, reflecting overall model performance across all classes. The curves were obtained by training on four folds and testing on the remaining fold, repeated five times. The final results represent the average across folds, providing a robust evaluation of generalizability and stability.

#### Classification results based on breakhis dataset

The BreakHis dataset contains images at four magnification levels, providing detailed categories and abundant pathological information. The proposed model achieved excellent performance on this dataset, with a test accuracy of 98.218%. It performed especially well at 100× and 200× magnifications, demonstrating strong discriminative power and stability. Table 8 summarizes the classification results across magnifications, while Table 9 reports metrics for the eight breast cancer subtypes.

**Table 8:** Classification results at four magnifications

Magnification	Acc (%)	Prec (%)	Rec (%)	F1 (%)
40×	97.75	97.80	97.75	97.76
100×	98.38	98.40	98.38	98.38
200×	99.12	99.14	99.12	99.12
400×	97.62	97.78	97.62	97.66

Magnification refers to the optical zoom level applied during image acquisition under a microscope, determining the spatial resolution of captured tissue structures. In the BreakHis dataset, histopathological images are collected at four magnification levels—40×, 100×, 200×, and 400×—where lower magnifications (e.g., 40×) provide a broader tissue context, while higher magnifications (e.g., 400×) reveal finer morphological details.

**Table 9:** Classification results of eight breast cancer pathology images

Sub Class	Acc (%)	Prec (%)	Rec (%)	F1 (%)
A	99.50	99.75	99.50	99.62
DC	98.25	93.98	98.25	96.02
F	97.25	98.25	97.25	97.74
LC	98.00	98.52	98.00	98.24
MC	98.00	98.74	98.00	98.37
PC	98.25	99.25	98.25	98.74
PT	97.75	99.25	97.75	98.49
TA	98.75	98.51	98.75	98.63

A, Adenosis; DC, Ductal Carcinoma; F, Fibroadenoma; LC, Lobular Carcinoma; MC, Mucinous Carcinoma; PC, Papillary Carcinoma; PT, Phyllodes Tumor; TA, Tubular Adenoma

**Fig. 7:**
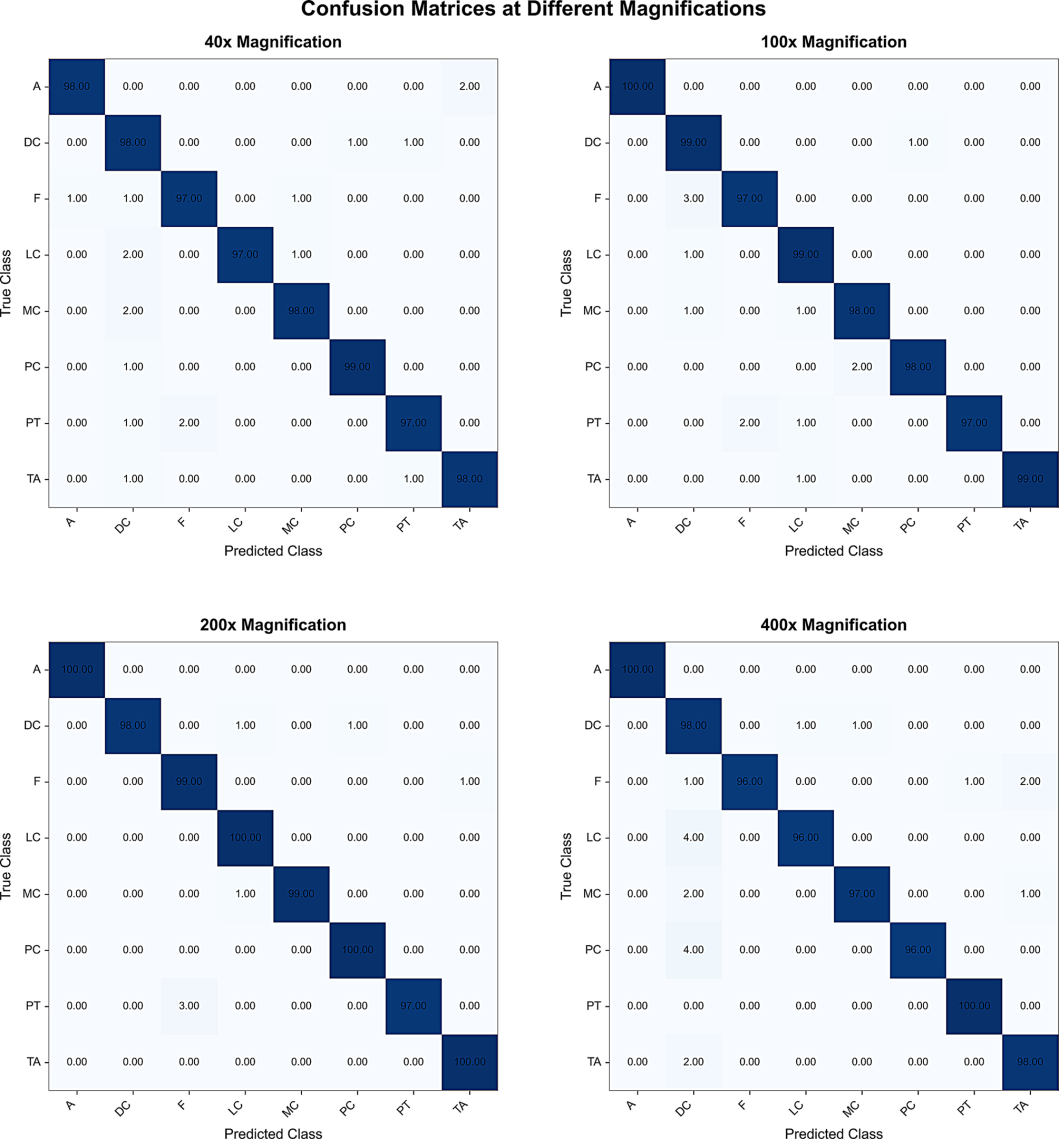
BreakHis-Confusion. The model demonstrates robust classification performance across eight tumor subtypes and four magnification levels, reflecting strong generalization and discriminative ability

Figure 7 shows the confusion matrices of the model on the BreakHis dataset, illustrating its classification performance across eight pathology categories at four magnification levels. To enrich the dataset and improve robustness, each image was divided into uniform patches for classification. Final predictions were generated by aggregating patch-level results through majority voting.

### Comparison results with advanced methods

We selected 11 baseline methods for comparison. Among them, DenseNet + FA + GuSA, TransMIL, and MA-MIDN adopt multi-instance learning strategies: DenseNet + FA + GuSA performs both lesion localization and image classification; TransMIL leverages a Transformer backbone to incorporate morphological and spatial information; and MA-MIDN introduces a novel multi-view attention (MVA) module to localize lesion regions. MSMV-PFENet integrates progressive feature coding with Bi-LSTM to extract multi-scale features, automatically focusing on cancer-related regions based on nuclear density. CTransNet uses DenseNet with ImageNet pretraining and a weighted feature fusion strategy. SwinCNN combines Swin Transformer and CNNs to exploit the strengths of both paradigms. DWNAT-Net incorporates Discrete Wavelet Transform (DWT) with Neighborhood Attention Transform (NAT) to jointly capture frequency and spatial information for improved accuracy.

To ensure fair comparison, all baseline models and our method were trained under a unified experimental setup. The same dataset partitions, preprocessing, and data augmentation strategies were used, together with a consistent optimization protocol: AdamW optimizer, cosine annealing learning rate with warmup, batch size of 32, and 50 training epochs. Grid search was applied to key hyperparameters (e.g., learning rate) to ensure optimal performance for each model. All experiments were conducted on an NVIDIA RTX 4090 GPU.

For statistical rigor, dataset splits were fixed, and each model was independently trained and evaluated 10 times with random seeds (0–9). Results are reported as mean ± standard deviation (SD), along with 95% confidence intervals (CI) to quantify precision. Statistical significance was assessed using paired t-tests and Cohen’s d effect sizes, with multiple comparisons corrected via the Benjamini–Hochberg procedure. This evaluation protocol ensures both robustness and fairness.

Table 10 below demonstrates the results of the quantitative comparative analysis of the models based on the BACH dataset, and the classification results of our model are significantly better than the other methods.

**Table 10:** Results of quantitative comparative analysis on the BACH dataset

Main network or method	Accuracy (mean ± SD, %)	Precision (mean ± SD, %)	Recall (mean ± SD, %)	F1 Score (mean ± SD, %)
TransMIL [27]	*85.83 ± 1.20	86.90 ± 1.35	84.69 ± 1.42	85.78 ± 1.30
CTransNet [28]	*88.75 ± 1.45	89.28 ± 1.58	88.75 ± 1.65	88.73 ± 1.55
EfficientNetV2	*89.53 ± 1.67	90.12 ± 1.78	88.94 ± 1.82	89.41 ± 1.74
DenseNet + FA + GuSA [29]	*90.25 ± 1.25	95.34 ± 1.10	85.57 ± 1.21	90.11 ± 1.26
ConvNeXt	*90.81 ± 1.45	91.23 ± 1.51	90.42 ± 1.52	90.72 ± 1.48
DWNAT-Net [30]	*91.25 ± 1.01	89.53 ± 1.09	92.89 ± 1.13	90.50 ± 1.07
FCCS-Net [31]	*91.25 ± 0.88	91.18 ± 0.92	91.37 ± 0.95	91.25 ± 0.93
Swin Transformer	*91.52 ± 1.05	92.15 ± 1.18	91.23 ± 1.25	91.32 ± 1.15
SwinCNN [15]	*92.89 ± 0.82	93.00 ± 0.85	91.40 ± 0.98	93.00 ± 0.80
MA-MIDN [22]	*93.57 ± 0.85	96.18 ± 0.80	94.26 ± 0.82	95.18 ± 0.79
MSMV-PFENet [22]	*94.80 ± 0.78	94.80 ± 0.70	94.80 ± 0.84	94.40 ± 0.75
Ours	96.40 ± 0.92	96.34 ± 0.97	96.36 ± 1.05	96.35 ± 1.12

Results marked with ‘*’ indicate statistically significant differences compared to our proposed baseline model, ResViT-GANNet. All comparisons: p-value < 0.01 for accuracy

**Fig. 8:**
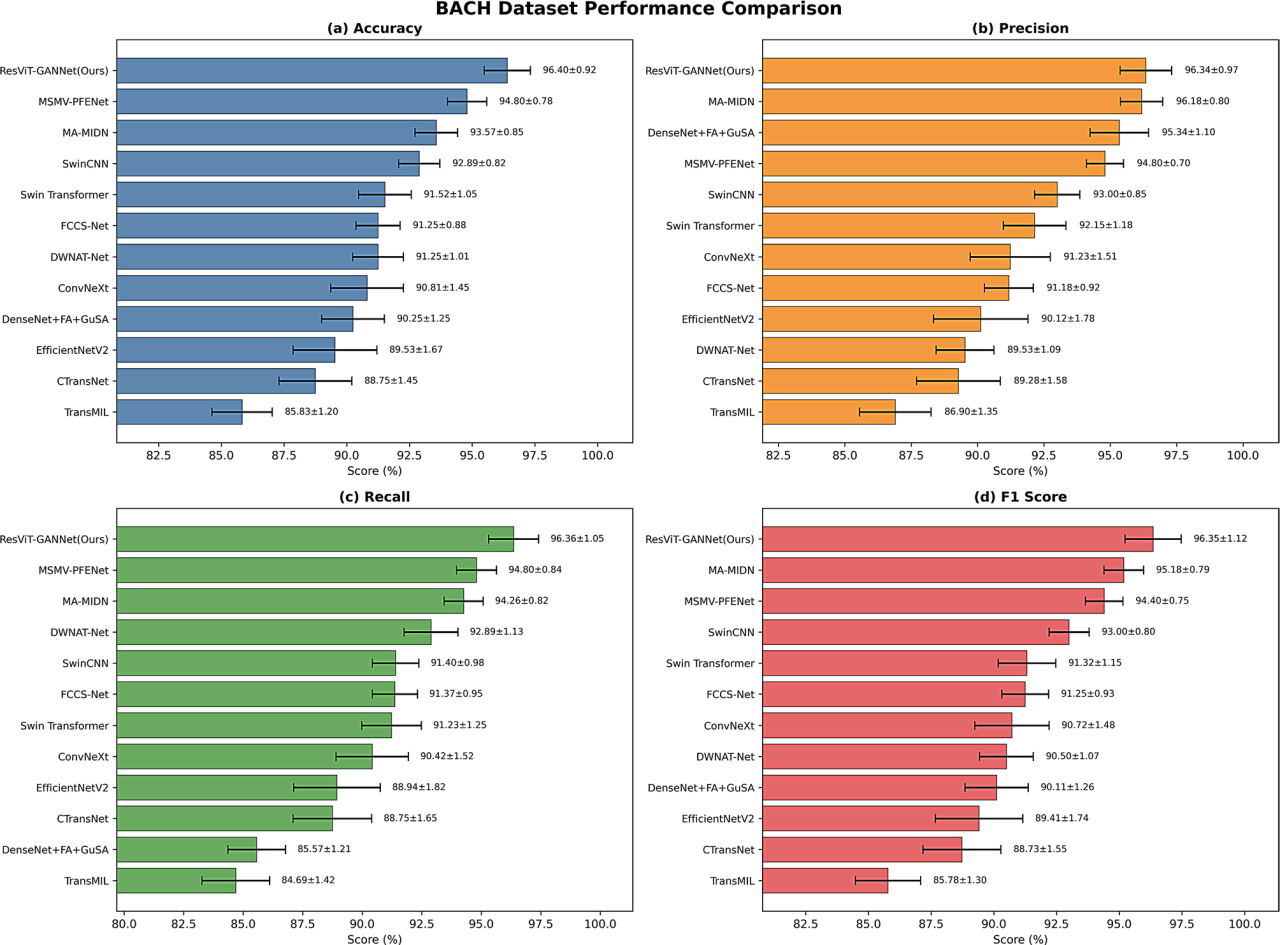
Performance comparison of different methods on the BACH dataset. Bar plots with error bars show mean ± SD values of four metrics: (**a**) Accuracy, (**b**) Precision, (**c**) Recall, and (**d**) F1 Score. Results are averaged across multiple runs, and ResViT-GANNet (Ours) consistently achieves superior performance compared with state-of-the-art methods

The quantitative superiority of ResViT-GANNet is vividly illustrated in Fig. 8, which provides a comprehensive visual comparison of all competing methods across the four key evaluation metrics on the BACH dataset. The bar plots with error bars clearly demonstrate that our model not only achieves the highest mean values in accuracy, precision, recall, and F1 score but also maintains consistently low standard deviations. This graphical representation reinforces the statistical robustness of ResViT-GANNet, supporting the claims derived from the numerical results in Table 10.

For the BreakHis dataset, we also selected 11 baseline methods for comparison. Table 11 below shows the quantitative comparative analysis results of the models based on the BreakHis dataset. The overall classification results of our model are better than those of other methods.

**Table 11:** Quantitative comparative analysis results on the BreakHis dataset

Main network or method	Magnification (mean ± SD, %)				Average accuracy (mean ± SD, %)
	40×	100×	200×	400×	
EfficientNetV2	91.54 ± 1.15	92.19 ± 1.08	92.07 ± 1.13	91.88 ± 1.16	*91.92 ± 0.58
ConvNeXt	92.78 ± 1.00	92.49 ± 1.05	93.23 ± 0.98	92.11 ± 1.08	*92.65 ± 0.51
Swin Transformer	93.56 ± 0.95	93.33 ± 0.98	94.09 ± 0.92	93.64 ± 0.96	*93.66 ± 0.48
BreaST-Net [23]	96.00 ± 1.10	92.60 ± 1.05	93.50 ± 1.00	93.40 ± 0.80	*93.88 ± 0.74
SE-Res-ConvNet [33]	96.00 ± 1.05	93.00 ± 0.85	98.00 ± 0.95	94.00 ± 0.92	*95.25 ± 0.93
DRDA-Net [34]	95.72 ± 0.96	94.41 ± 1.08	97.43 ± 1.12	98.10 ± 0.98	*96.42 ± 0.78
VTHSC-MIR [35]	97.20 ± 0.75	97.60 ± 0.70	97.10 ± 0.72	98.20 ± 0.68	*97.53 ± 0.38
5-B Network [36]	98.10 ± 0.70	97.40 ± 0.75	97.50 ± 0.74	97.20 ± 0.76	*97.55 ± 0.38
BreastNet [37]	97.99 ± 0.65	97.84 ± 0.68	98.51 ± 0.60	95.88 ± 0.85	*97.56 ± 0.55
CoatNet [38]	96.83 ± 0.72	98.40 ± 0.65	97.52 ± 0.69	97.80 ± 0.70	*97.64 ± 0.35
AHoNet [39]	97.58 ± 0.68	97.47 ± 0.70	99.09 ± 0.55	96.52 ± 0.78	*97.67 ± 0.54
Ours	97.75 ± 0.74	98.38 ± 0.82	99.12 ± 0.78	97.62 ± 0.62	98.22 ± 0.37

Results marked with ‘*’ indicate statistically significant differences compared to our proposed baseline model, ResViT-GANNet. All comparisons: p-value < 0.01 for average accuracy

**Fig. 9:**
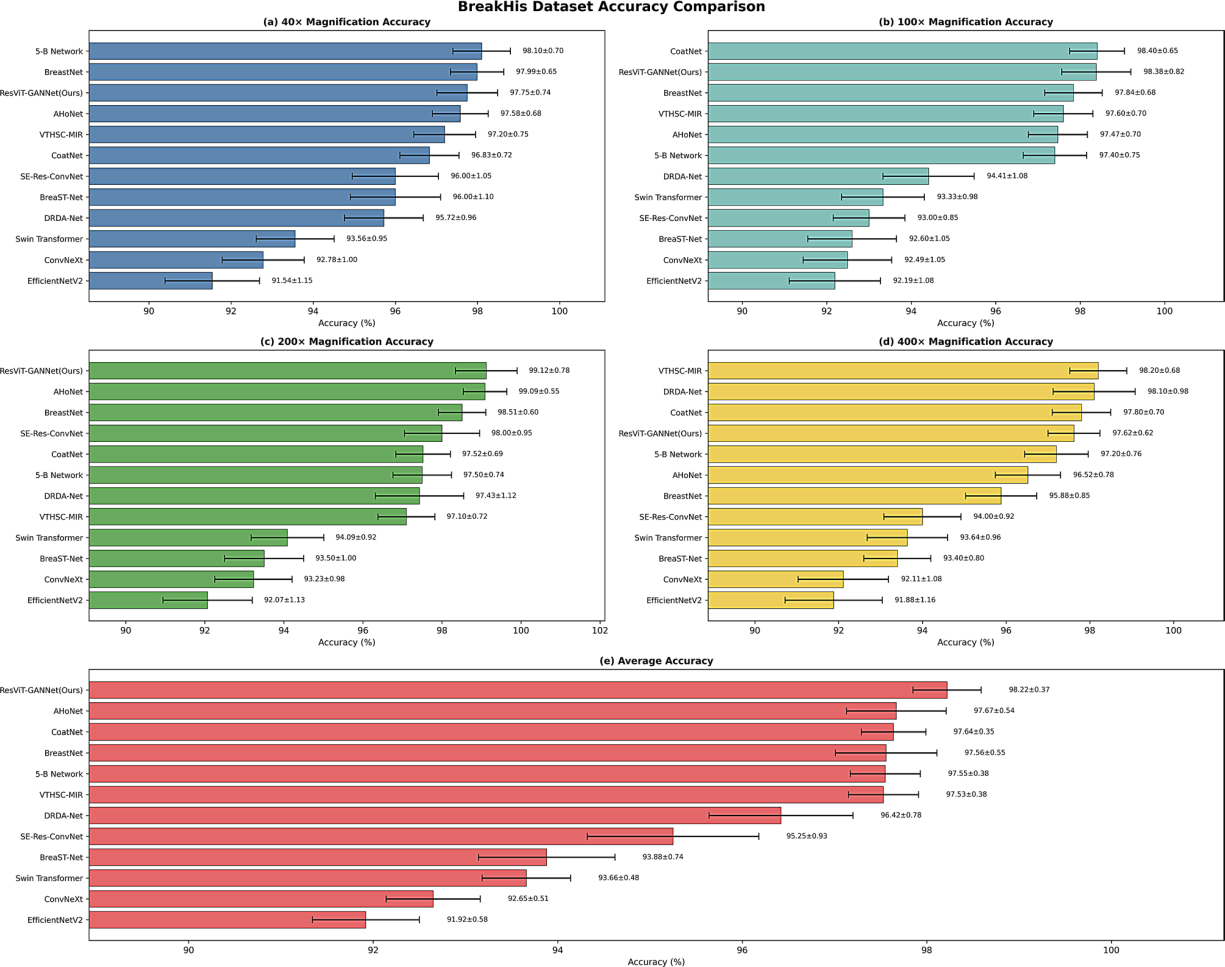
Accuracy comparison of different methods on the BreakHis dataset under various magnifications. Bar plots with error bars show mean ± SD values at (**a**) 40×, (**b**) 100×, (**c**) 200×, and (**d**) 400× magnification, as well as (**e**) the average accuracy across all magnifications. ResViT-GANNet (Ours) demonstrates consistently higher accuracy and robustness compared with competing methods

To further evaluate performance across varying microscopic resolutions, Fig. 9 presents the classification accuracy of all baseline methods and our proposed ResViT-GANNet at each magnification level (40×, 100×, 200×, 400×) as well as the overall average on the BreakHis dataset. The results show that our model sustains consistently high accuracy across all magnifications, with a particularly notable peak at 200×. This visual evidence confirms that the architectural advantages of ResViT-GANNet remain effective regardless of image scale, thereby achieving state-of-the-art average accuracy and demonstrating exceptional generalization capability—an essential requirement for reliable clinical deployment under diverse imaging conditions.

In addition to reporting accuracy and related metrics, we further quantified the practical significance of performance differences using Cohen’s *d* effect size, calculated from ten independent runs per model. As summarized in Table 12, our proposed method achieved Cohen’s *d* values consistently above 1.0. According to conventional benchmarks (Cohen, 1988), these values indicate large effect sizes, suggesting that the performance improvement is not only statistically significant (*p* < 0.01), but also of substantial practical relevance in clinical applications.

**Table 12:** Cohen’s d effect sizes for accuracy comparison on BACH and BreakHis datasets

	Models	Average accuracy (d) ↑
BACH	TransMIL	1.01
	CTransNet	1.16
	EfficientNetV2	1.12
	DenseNet + FA + GuSA	1.34
	ConvNeXt	1.18
	DWNAT-Net	1.25
	FCCS-Net	1.21
	Swin Transformer	1.20
	SwinCNN	1.35
	MA-MIDN	1.27
	MSMV-PFENet	1.17
BreakHis	EfficientNetV2	1.15
	ConvNeXt	1.21
	Swin Transformer	1.32
	BreaST-Net	1.24
	SE-Res-ConvNet	1.07
	DRDA-Net	1.19
	VTHSC-MIR	1.37
	5-B Network	1.37
	BreastNet	1.48
	CoatNet	1.14
	AHoNet	1.15

Cohen’s *d* was computed based on ten independent experimental runs per model. All values greater than 1.0 indicate large effect sizes, demonstrating substantial performance gains achieved by our method compared to baselines.

### Qualitative analysis with Grad-CAM visualizations

**Fig. 10:**
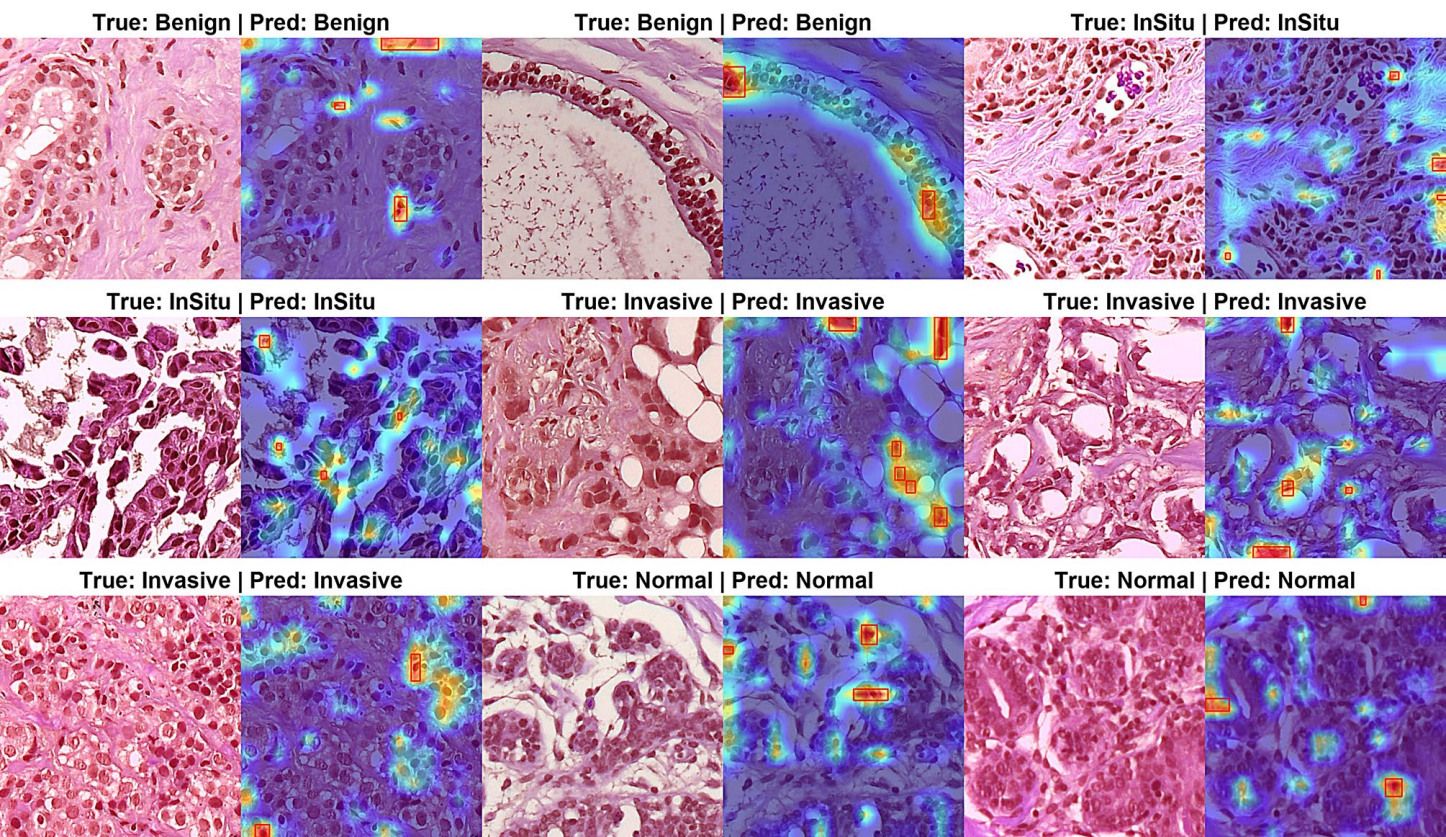
Correct Prediction.Activation heatmaps highlight lesion-relevant regions attended by the model, demonstrating its capability to localize diagnostically significant areas across multiple breast cancer subtypes

As shown in Fig. 10, the Grad-CAM visualizations confirm that the model’s predictions are driven by attention to diagnostically significant tissue structures. The heatmaps demonstrate a strong alignment between the model’s focused regions and pathologically critical areas, validating the clinical relevance of its decision-making process.

Activation heatmaps highlight lesion-relevant regions attended by the model, demonstrating its capability to localize diagnostically significant areas across multiple breast cancer subtypes. (a) Invasive Carcinoma: The model correctly focuses on regions exhibiting high nuclear density, pleomorphism, and stromal invasion patterns. (b) Ductal Carcinoma In Situ (DCIS): Attention is localized to involved ductal structures with characteristic monomorphic cell populations and polarized mitosis. (c) Benign Tumors: The activation maps cover well-organized glandular formations with maintained polarity and absence of significant atypia. (d) Normal Tissue: The model attends to normal lobular architecture and uniform cell distribution, correctly ignoring irrelevant adipose or stromal regions.

This consistent behavior across subtypes indicates that the model has effectively learned to prioritize histopathological features that are definitive for diagnostic differentiation. The precision in localizing these features—such as ignoring non-diagnostic background in normal tissue and highlighting malignant nuclei in carcinomas—underscores the model’s ability to emulate pathological reasoning and explains its high classification accuracy.

**Fig. 11:**
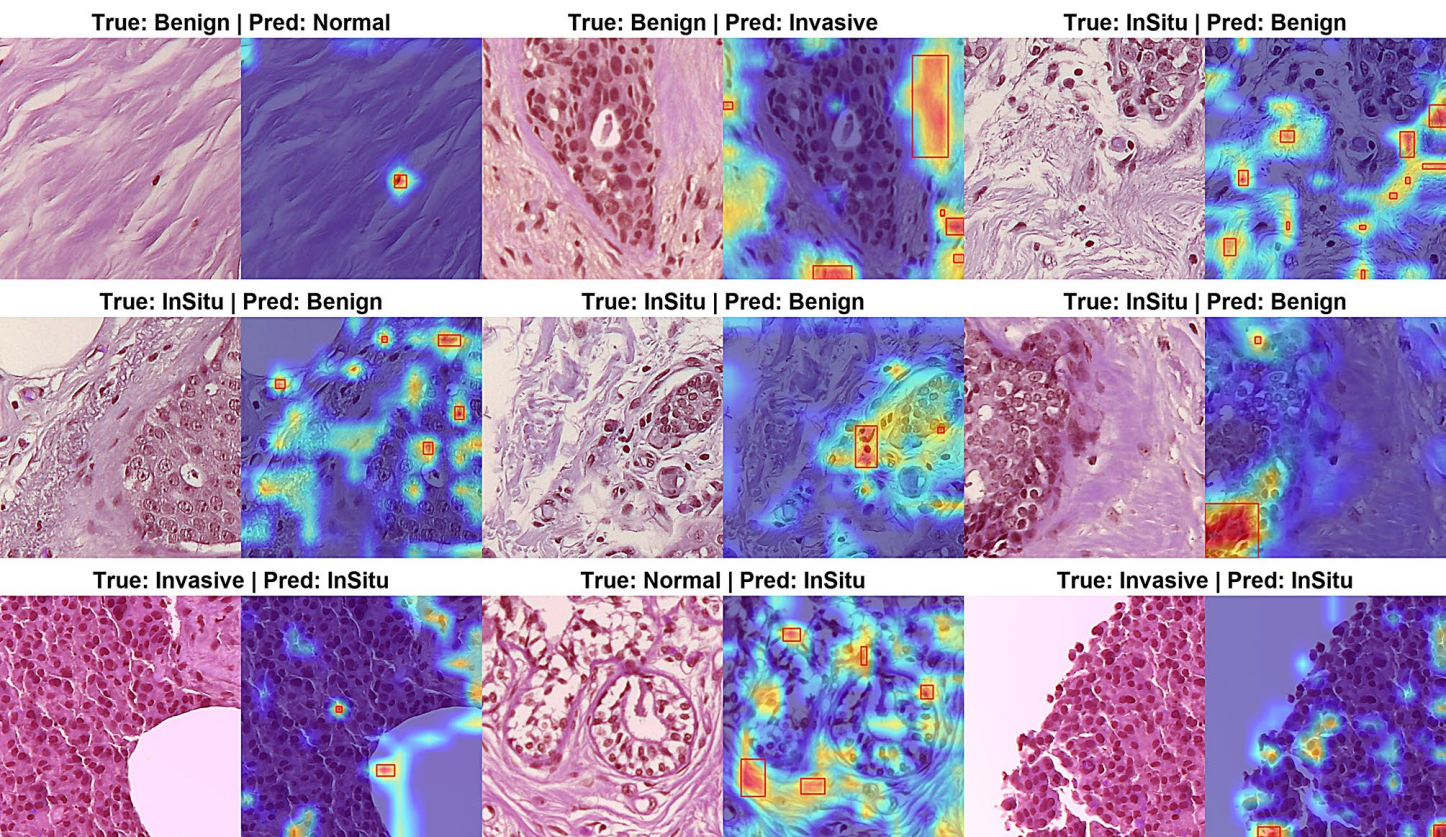
Wrong Prediction.The model predominantly attends to ambiguous or non-discriminative regions, providing insights into potential causes of prediction errors and classification confusion

Figure 11 presents an analysis of the model’s misclassification patterns, which offers actionable insights for enhancing the model’s classification performance. Our analysis of failure cases reveals that misclassifications primarily occur between morphologically similar categories. Key patterns include:

Benign vs. Normal: The model sometimes misclassifies benign lesions as normal tissue due to their well-organized cellular structure and minimal nuclear atypia, particularly in low-magnification regions.

InSitu vs. Benign: Well-differentiated DCIS cases with preserved architecture are occasionally confused with benign proliferative lesions, as the model may overlook subtle malignant features such as monomorphic cell populations and polarized mitosis.

Invasive vs. InSitu: Early invasive carcinomas with well-defined glandular structures or limited stromal invasion are sometimes misclassified as in situ lesions, especially when invasive foci are small or lack prominent stromal response.

Normal vs. InSitu: Dense normal lobules exhibiting cellular crowding may be misinterpreted as low-grade DCIS, reflecting a known diagnostic challenge even in human pathology.

These errors typically occur in regions with ambiguous glandular patterns, low contrast, or technical artifacts (as highlighted by red boxes in Fig. 5). The confusions stem primarily from inherent morphological similarities between categories and the model’s difficulty in recognizing subtle diagnostic features that require high-resolution context or additional immunohistochemical staining.

Future work will address these limitations through enhanced attention mechanisms and incorporation of additional clinical context to improve differentiation of morphologically similar lesions.

### Ablation experiments

In order to validate the actual contribution of the proposed improved modules to the model classification performance, we designed a series of ablation experiments by gradually removing or replacing key components of the model for comparison. Specifically, they include evaluating the effectiveness of the modified ResNet branch, the ViT branch, the multimodal attention mechanism, and the TA Layer and data generation. To ensure the scientific validity of the experiments and the fairness of the comparisons, we use the standard ResNet network as the baseline model and the BACH dataset as an example for control experiments. Each model configuration was trained and evaluated under the same training conditions, the classification performance metrics for each ablation experiment are summarized in Table 13 below.

**Table 13:** Comparison of ablation results

Model	Accuracy	Precision	Recall	F1 Score
ResNet	*87.019	87.114	87.047	86.983
ResNet + SE	*89.740	89.649	89.734	89.648
ViT	*89.523	90.480	89.540	90.426
ResNet + ViT	*85.446	85.427	85.300	85.218
ResNet + ViT + MultiModalAttention + TA Layer	*90.141	90.162	90.153	90.021
ResNet + SE + ViT + MultiModalAttention + TA Layer	*93.149	93.169	93.085	93.080
ResNet + SE + ViT + MultiModalAttention + TA Layer + Data generation(Our Model)	96.403	96.341	96.364	96.347

Results marked with ‘*’ are statistically significantly different from ResViT-GANNet (*p* < 0.01, Cohen’s d > 1.0 for accuracy)

### Limitations of the experimental study

It is important to acknowledge several limitations inherent in our experimental design and dataset handling, which could influence the interpretation of the results.

First, for the BACH dataset, the lack of patient identifiers required an image-level split, which carries a potential risk of overestimating generalization performance if patches from the same patient are distributed across different sets.

Second, despite employing patient-level splitting for BreakHis, significant class imbalance persists. Performance on underrepresented subtypes (e.g., Lobular Carcinoma) may be less reliable than on prevalent classes (e.g., Ductal Carcinoma).

Third, while our evaluation metrics show strong overall performance, they may conceal specific failures on morphologically ambiguous cases that require more nuanced analysis.

Fourth, our hyperparameter selection was guided by empirical validation and ablation studies, but not by exhaustive search. Different configurations might yield additional improvements.

Finally, the preprocessing approaches—fixed grid patching for BACH and overlapping patches for BreakHis—represent a trade-off between computational efficiency and optimal diagnostic coverage, which may influence model performance.

These limitations reflect common challenges in computational pathology, and we have sought to address them through robust validation practices. Nevertheless, they should be considered when interpreting our results.

## Discussion

### Key contributions and innovations

This study presents a novel dual-branch network (ResViT-GANNet) that integrates ResNet and Vision Transformer architectures, marking a significant advancement in breast cancer histopathological image classification. Our approach introduces three key innovations: (1) a parallel dual-branch architecture with a novel Token-Aligned Multimodal Attention (TAMA) fusion module, which enables a deep, context-aware alignment of heterogeneous features, effectively overcoming the irreversible feature loss common in serial hybrid models; (2) pathology-specific feature optimization, incorporating channel recalibration (SE) in the CNN branch to emphasize tumor-relevant regions and multi-layer [CLS] token fusion in the ViT branch to capture hierarchical cellular-to-tissue-level semantics, thereby mirroring pathologists’ diagnostic reasoning; and (3) a holistic framework integrating StyleGAN2-ADA-based generative augmentation and Grad-CAM interpretability, which simultaneously addresses data scarcity and enhances clinical trust. Unlike conventional single-stream or shallow fusion approaches, our method simultaneously captures fine-grained local pathological details and robust global semantic information—both critical for precise subtype discrimination.

### Theoretical justification

The superior performance of ResViT-GANNet can also be explained mathematically.

1. **Complementary representations**: The CNN branch models local morphological details via convolution $$\:{\varvec{F}}_{\varvec{c}\varvec{n}\varvec{n}}=\varvec{W}\:\times\:\varvec{X}+\varvec{b}$$, while the Transformer branch captures global contextual relations via attention softmax$$\:\left(\raisebox{1ex}{${\varvec{Q}\varvec{K}}^{\varvec{T}}$}\!\left/\:\!\raisebox{-1ex}{$\sqrt{\varvec{d}}$}\right.\right)V$$. Their fusion ensures integration of high-frequency local features and low-frequency global semantics.

38 $$\:{\varvec{F}}_{\varvec{f}\varvec{u}\varvec{s}\varvec{i}\varvec{o}\varvec{n}}=\varvec{\alpha\:}{\varvec{F}}_{\varvec{c}\varvec{n}\varvec{n}}+\varvec{\beta\:}{\varvec{F}}_{\varvec{v}\varvec{i}\varvec{t}}\:\:\:\:\:\:\varvec{\alpha\:},\varvec{\beta\:}\varvec{ϵ}\mathbb{R}\mathbb{\:}\mathbb{\:}\mathbb{\:}$$

2. **Information-theoretic advantage of TAMA**: By applying cross-modal attention, TAMA maximizes the mutual information ***I***($\:{F}_{cnn};{F}_{vit}$), reducing redundancy and preserving complementarity.
3. **Generalization bound improvement**: GAN-based augmentation narrows the gap between empirical risk $\:\widehat{R}\left(f\right)$and true risk *R(f)*, thus reducing generalization error.
4. **Comparison with baselines**: Unlike CNN-only (limited receptive fields), Transformer-only (loss of fine details), or serial CNN–Transformer hybrids (irreversible feature compression), our parallel dual-branch design preserves both fine-grained and global features.

This theoretical justification clarifies why our network consistently outperforms baseline models.

### Performance gains and clinical relevance

To address the prevalent challenges of limited medical datasets, we incorporated a StyleGAN2-ADA-based synthetic image generation module. Our results demonstrate that this augmentation strategy contributed to an average accuracy improvement of 3.3%, effectively enhancing the model’s generalization capability and robustness, and overcoming a major bottleneck in histopathology image analysis. The seamless integration of generative augmentation with the dual-branch fusion framework constitutes a novel methodology not previously explored in breast cancer pathology classification.

Clinically, the high accuracy and robustness of ResViT-GANNet translate into tangible benefits. The model can serve as a powerful decision-support tool for pathologists, particularly in distinguishing morphologically similar subtypes (e.g., Benign vs. InSitu) and in screening scenarios with high workload, potentially reducing diagnostic variability and improving throughput. The statistically significant improvements (*p* < 0.01) with large effect sizes (Cohen’s d > 1.0) underscore that these advances are not merely algorithmic but carry substantial practical relevance. Furthermore, the model’s interpretability, enhanced through Grad-CAM visualizations, provides transparent insights into the key histological regions (e.g., ductal structures, nuclear pleomorphism) influencing predictions. This transparency is crucial for fostering clinical trust and facilitating the practical adoption of AI tools in diagnostic workflows.

### Limitations and generalizability

Despite these strengths, our study has limitations. First, the increased computational complexity inherent in the dual-branch architecture presents challenges for real-time clinical application on standard hardware. Future research should focus on model compression and optimization techniques, such as knowledge distillation or pruning, to enhance feasibility in routine clinical workflows. Second, while StyleGAN2-ADA mitigates data scarcity, our training data were sourced from public datasets (BACH, BreakHis). The model’s performance must be further validated on multi-center, prospective cohorts encompassing a wider range of scanners, staining protocols, and population diversity to confirm its robustness against real-world variability. Third, although Grad-CAM improves interpretability, it remains a post-hoc explanation method; its absolute reliability in all clinical scenarios requires further investigation.

Regarding generalizability, the model demonstrated strong performance on two distinct public datasets involving different classification tasks (4-class and 8-class) and imaging magnifications. The consistent superiority over state-of-the-art methods across these datasets suggests a robust feature representation capability. However, the ultimate test of generalizability lies in external validation. Future work must include extensive external validation across diverse, independent clinical cohorts and histopathology imaging protocols to firmly establish the model’s efficacy and reliability before broad clinical translation can be considered.

In conclusion, the ResViT-GANNet framework represents a comprehensive solution that integrates architectural innovation, data augmentation, and explainable AI. By addressing key technical and clinical challenges, our study provides a solid foundation for developing reliable AI-assisted diagnostic systems in breast cancer pathology.

## Conclusions

In this study, we introduced ResViT-GANNet, a novel dual-branch deep learning framework for breast cancer histopathological image classification. The design synergistically integrates a CNN branch for fine-grained local feature extraction with a Transformer branch for global contextual modeling, thereby overcoming the limitations of conventional serial hybrids. Two additional contributions strengthen the framework: the Token-Aligned Multimodal Attention (TAMA) module for effective feature fusion, and a StyleGAN2-ADA-based generative augmentation strategy that mitigates data scarcity while enhancing interpretability through Grad-CAM visualization.

Extensive experiments on the BACH and BreakHis datasets confirm that ResViT-GANNet achieves state-of-the-art performance, offering a robust, accurate, and interpretable solution that aligns with practical diagnostic needs. By jointly addressing feature fusion, data imbalance, and transparency, this work contributes a significant step toward trustworthy AI-assisted pathology.

## Future directions

While ResViT-GANNet shows promising results, we identify several exciting avenues for future research to further advance its clinical translation and capabilities:

Model Efficiency Optimization: The computational overhead of the dual-branch architecture currently limits real-time application. Future work will focus on model compression techniques, such as knowledge distillation and neural network pruning, to develop a lighter-weight variant without significantly compromising performance.

Large-Scale Multi-Center Validation: To unequivocally establish generalizability and robustness, a rigorous external validation across multiple independent medical institutions, encompassing a wider variety of scanners, staining protocols, and patient demographics, is essential as a next step.

Extension to Other Diagnostic Tasks: We plan to extend the core principles of ResViT-GANNet beyond classification to other critical histopathology tasks, such as nuclei segmentation, tumor microenvironment analysis, and prediction of molecular subtypes or patient prognosis.

Development of Human-in-the-Loop Systems: Ultimately, for seamless clinical integration, we envision developing interactive diagnostic systems where the model’s predictions and visual explanations serve as an intuitive “second opinion” tool within the pathologist’s workflow, fostering collaboration between AI and human expertise.

## Data Availability

The datasets used in this study, BACH and BreakHis, are publicly accessible through their respective open-access repositories. Processed data and code supporting the findings of this study are available from the corresponding author upon reasonable request.

## Abbreviations

Grad-CAM: Gradient-weighted Class Activation Mapping
CNNs: Convolutional Neural Networks
ViT: Vision Transformer
TA: Token Alignment
SE: Squeeze-and-Excitation
MHSA: Multi-Head Self-Attention
AMP: Automatic Mixed Precision Training
ACC: Accuracy
Prec: Precision
Rec: Recall
F1: F1 score
